# Multigene phylogeny of the Mustelidae: Resolving relationships, tempo and biogeographic history of a mammalian adaptive radiation

**DOI:** 10.1186/1741-7007-6-10

**Published:** 2008-02-14

**Authors:** Klaus-Peter Koepfli, Kerry A Deere, Graham J Slater, Colleen Begg, Keith Begg, Lon Grassman, Mauro Lucherini, Geraldine Veron, Robert K Wayne

**Affiliations:** 1 Department of Ecology and Evolutionary Biology, University of California, Los Angeles, CA, 90095-1606, USA; 2 Postnet Suite 230, Private Bag X18, Rondebosch, 7701, Republic of South Africa; 3 Caesar Kleberg Wildlife Research Institute, MSC 218, 700 University Boulevard, Texas A and M University-Kingsville, Kingsville, TX 78363, USA; 4 Grupo de Ecología Comportamental de Mammiferos (GECM), Cátedra Fisiología Animal, Departamento de Biología, Bioquímica y Farmacia, Universidad Nacional del Sur – CONICET, San Juan 670, 8000 Bahía Blanca, Argentina; 5 Muséum National d'Histoire Naturelle, Département Systématique et Evolution, CP 51 USM 601-UMR 5202, Origine, Structure et Evolution de la Biodiversité, 57 Rue Cuvier, 75231 Paris Cedex 05, France

## Abstract

**Background:**

Adaptive radiation, the evolution of ecological and phenotypic diversity from a common ancestor, is a central concept in evolutionary biology and characterizes the evolutionary histories of many groups of organisms. One such group is the Mustelidae, the most species-rich family within the mammalian order Carnivora, encompassing 59 species classified into 22 genera. Extant mustelids display extensive ecomorphological diversity, with different lineages having evolved into an array of adaptive zones, from fossorial badgers to semi-aquatic otters. Mustelids are also widely distributed, with multiple genera found on different continents. As with other groups that have undergone adaptive radiation, resolving the phylogenetic history of mustelids presents a number of challenges because ecomorphological convergence may potentially confound morphologically based phylogenetic inferences, and because adaptive radiations often include one or more periods of rapid cladogenesis that require a large amount of data to resolve.

**Results:**

We constructed a nearly complete generic-level phylogeny of the Mustelidae using a data matrix comprising 22 gene segments (~12,000 base pairs) analyzed with maximum parsimony, maximum likelihood and Bayesian inference methods. We show that mustelids are consistently resolved with high nodal support into four major clades and three monotypic lineages. Using Bayesian dating techniques, we provide evidence that mustelids underwent two bursts of diversification that coincide with major paleoenvironmental and biotic changes that occurred during the Neogene and correspond with similar bursts of cladogenesis in other vertebrate groups. Biogeographical analyses indicate that most of the extant diversity of mustelids originated in Eurasia and mustelids have colonized Africa, North America and South America on multiple occasions.

**Conclusion:**

Combined with information from the fossil record, our phylogenetic and dating analyses suggest that mustelid diversification may have been spurred by a combination of faunal turnover events and diversification at lower trophic levels, ultimately caused by climatically driven environmental changes. Our biogeographic analyses show Eurasia as the center of origin of mustelid diversity and that mustelids in Africa, North America and South America have been assembled over time largely via dispersal, which has important implications for understanding the ecology of mustelid communities.

## Background

"Wave after wave of immigration came in from Asia, recruiting the fauna at each successive stage, but leaving little opportunity for new types to arise here. Even those genera which seem to be of native origin, might prove to be immigrants, if all their history were known ([1], p 593)."

The diversification of the Mustelidae (Carnivora, Mammalia) is a striking example of adaptive radiation, the evolution of ecological and phenotypic diversity from a common ancestor [2]. Mustelids exhibit both locomotor and dietary diversity, with taxa that are fossorial (badgers), semi-arboreal (martens) and semi-aquatic (otters), and diets that vary from specialization on rodents (weasels) to piscivory (otters). Ecomorphological diversity in the family is thus correspondingly high, reflecting the adaptation of different species of mustelids to different habits and habitats. As with other cases of adaptive radiation [3], resolving relationships within the Mustelidae, especially among genera, has been challenging. Many taxonomic schemes proposed for mustelids within the last century were based on morphology and classified genera into various numbers of subfamilies [4,5] whose boundaries were largely determined by ecomorphological similarity. At one extreme, Pocock [4] divided extant mustelids into 15 (mostly monotypic) subfamilies based on descriptive analyses of external characters (e.g. structure of the rhinarium and feet). At the other end, the system proposed by Simpson [5] cast mustelids into five subfamilies based on both phylogeny and 'similarity in adaptiveness' of constituent genera: Lutrinae (otters), Melinae (badgers), Mellivorinae (honey badger), Mephitinae (skunks) and Mustelinae (martens and weasels). While such a scheme may indeed reflect the true phylogeny in some instances, morphological similarity does not necessarily imply phylogenetic affinity, as has been well demonstrated in certain groups that exemplify adaptive radiation (e.g. *Anolis *lizards [6]). Moreover, such criteria can lead to recognition of polyphyletic grades rather than monophyletic groups. Nonetheless, Simpson's subfamilial classification of the Mustelidae has been followed for many years, although the latest classification provisionally places all mustelids (excluding skunks and stink badgers) into two subfamilies, Lutrinae and Mustelinae [7]. This latter scheme was proposed in recognition of demonstrated paraphyly of the traditional subfamilies by more recent morphological-based phylogenetic studies [8].

During the last decade, DNA sequence-based studies have begun to challenge the validity of the five-subfamily scheme and even monophyly of the family itself. Studies using both mitochondrial and nuclear sequences have consistently demonstrated that skunks and stink badgers (*Mydaus*) descend from a common ancestor and together form a lineage (Mephitidae) that diverged prior to the split between Mustelidae and Procyonidae [9-11]. These studies and those more focused on mustelids [12-17] have also suggested that: (i) the Lutrinae is monophyletic; but that (ii) both Melinae and Mustelinae are polyphyletic. However, phylogenetic relationships within the family remain uncertain or unknown because taxon sampling, especially for genera, has been incomplete. Further, adequate character sampling is also an important issue because adaptive radiations are often composed of lineages that have rapidly diverged [2,3]. Short stem lengths of topologies revealed in recent studies, albeit with incomplete taxon sampling [11,13] suggests that deeper lineages of mustelids may have radiated within a short span of time. Adequate character sampling is therefore critical in achieving an accurate, well-resolved and robust phylogenetic hypothesis.

Fossil evidence indicates that the biogeographic history of mustelids is characterized by numerous intercontinental dispersals, primarily originating from Eurasia where the earliest fossil remains of the family (of Late Oligocene age) are found [18,19]. For instance, a large majority of mustelid diversity in North (and South) America is considered to have originated from lineages that repeatedly dispersed from Eurasia via the Bering land bridge [19,20]. The earliest immigrants to North America arrived in the Early Miocene and included a paraphyletic group of stem taxa referred to as 'paleomustelids', whose affinities to crown group mustelids ('neomustelids') remains ambiguous, as well as genera that belonged to the extinct subfamily Leptarctinae [20-25]. The first appearance of various mustelid genera in North America is used to help define the beginning of biostratigraphic boundaries of North American land mammal ages (NALMAs) during the Neogene [19,26]. For example, the first appearance of extinct genera *Trogonictis *and *Sminthosinis *and extant genera *Lutra *and *Mustela *help mark the latest Hemphilian NALMA (Late Miocene-Early Pliocene, 5.9-4.7 million years ago (MYA)) [19]. There is still uncertainty, however, about the exact number of intercontinental dispersal events underlying the biogeographic distribution of extant genera and species and, therefore, how much of mustelid continental diversity is a result of *in situ *versus *ex situ *evolution. Further, the sequence of dispersal events has been difficult to decipher, stemming from incompleteness of the fossil record. Clarifying the biogeographic history of mustelids has implications for understanding the community ecology of mustelids. Multiple species of mustelid are often found in the same community and consequently they have been the subject of important studies on character displacement and resource partitioning [27-29]. However, many of these studies lacked a historical perspective (via phylogeny), which has been shown to exert a strong influence on community assembly and structure [30,31].

Here, we present a nearly complete generic-level phylogeny of the Mustelidae using ~12,000 base pairs (bp) of mitochondrial and nuclear DNA data obtained from 22 gene segments. We use this phylogeny to address three objectives. First, we compare our phylogenetic hypothesis with previous hypotheses generated with morphological or molecular data. Second, we estimate relative divergence times using new Bayesian relaxed molecular clock methods to understand the temporal pattern of diversification in the family. Do mustelids exhibit one or more bursts of rapid cladogenesis characteristic of many adaptive radiations [2]? Moreover, correlation of divergence times with paleoenvironmental changes can provide insight into the mechanisms that might have been responsible for bursts of diversification [32]. Finally, we assess the biogeographic history of the Mustelidae, especially with regard to understanding dispersal history between continents of the Old World (Africa and Eurasia) and those of the New World (North and South America).

## Results and discussion

### Phylogenetic relationships

**Figure 1:**
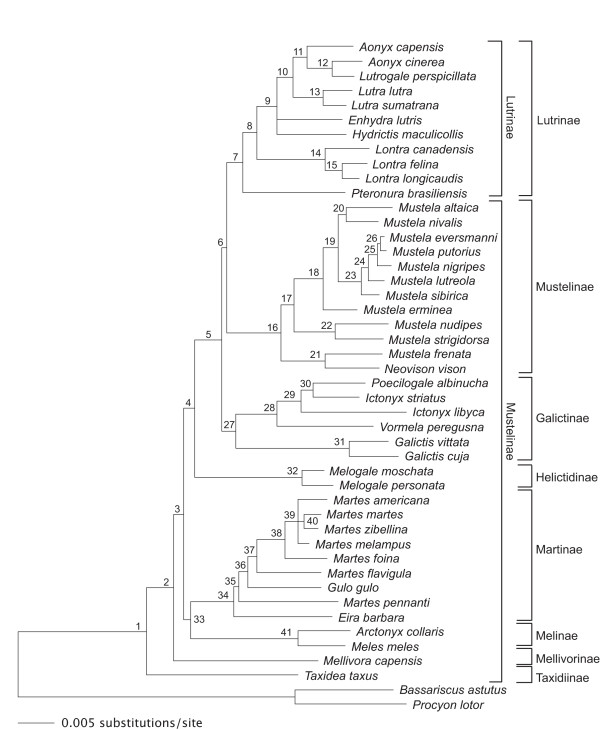
**Bayesian consensus phylogram of 14,002 trees (burn-in of 6,000 trees) for the Mustelidae using the GTR + I + G model of DNA substitution**. Nodes are numbered (1–41), with bootstrap (ML and MP) and posterior probabilities (BI) in Table 1. Brackets at right show subfamily classification as proposed by Wozencraft [7] (inside) and Fulton and Strobeck [11] and Sato et al [15] (outside). Branch lengths are proportional to number of substitutions per site (scale bar).

Phylogenetic analyses using maximum parsimony (MP), two different methods of maximum likelihood (ML) and Bayesian inference (BI) all recovered the same hypothesis for intergeneric relationships, which resolves Mustelidae into seven primary divisions that include four major clades and three monotypic lineages (Figure 1). Otters (*Aonyx*, *Enhydra*, *Hydrictis*, *Lontra*, *Lutra*, *Lutrogale *and *Pteronura*) form a clade (node 7) that is sister to a clade comprising mink and true weasels (*Mustela *and *Neovison*; node 16). These clades, in turn, are sister to a clade that includes weasel-like species with aposematically colored pelage (*Galictis*, *Ictonyx*, *Poecilogale *and *Vormela*; node 27). Next, ferret-badgers (*Melogale*) are a monotypic lineage (node 32) that is sister to these three combined clades. The fifth major clade (node 33) comprises two subclades, one containing hog-nosed and Eurasian badgers (*Arctonyx *and *Meles*) and the other containing tayra, wolverine and martens (*Eira*, *Gulo *and *Martes*). Finally, as the earliest divergences in the tree, the American badger (*Taxidea*) and honey badger (*Mellivora*) form successive monotypic lineages sister to all other mustelid genera. Intergeneric relationships observed are largely congruent with those recovered in recent analyses of mitochondrial and nuclear DNA sequences [10,11,13-17]. In contrast, our topology is highly incongruent with a cladistic analysis based on morphology [8]. For example, Bryant et al [8] found that *Melogale *was reconstructed as sister to all remaining mustelids and that *Eira*, *Gulo *and *Martes *were polyphyletic. Except for the monophyly of otters, skunks (including *Mydaus*), and *Arctonyx *and *Meles*, all other nodes in the Bryant et al [8] tree had low bootstrap support values (<50%). Furthermore, alternative topologies were recovered in the Bryant et al study when certain characters were weighted differentially.

Regarding relationships within the four major clades, otters are resolved into three primary lineages, whose relationships are congruent with previous analyses based on fewer DNA sequence data [12,13]: one containing Old World river otters and the sea otter (*Aonyx*, *Lutrogale*, *Lutra*, *Enhydra *and *Hydrictis*; node 9), a second containing New World river otters (*Lontra*; node 14) and a third containing the monotypic giant otter (*Pteronura*). Relationships of *Enhydra *and *Hydrictis *were unresolved in the Bayesian consensus tree (Figure 1), but were resolved differently in MP and ML analyses. With MP, *Enhydra *and *Hydrictis *were resolved as sister taxa, whereas with ML, they were resolved as successive sister lineages to remaining Old World otters, with *Hydrictis *forming the first basal split. However, neither of these relationships was well supported (<50% MP and ML bootstrap). Two clades comprise the true weasel and mink clade (*Mustela *and *Neovison*), with the New World American mink (*N. vison*) and long-tailed weasel (*M. frenata*) in one clade (node 21) and all other sampled *Mustela *species in the second clade (node 17). Within the latter clade, *M. nudipes *and *M. strigidorsa *are sister to a clade comprising species largely distributed in temperate regions of the northern hemisphere. Despite the similar ecology, American mink (*N. vison*) and European mink (*M. lutreola*) are distantly related, as found in previous studies [14,33,34]. Species of true weasels and mink have been divided into five [34] or nine subgenera [35] based on morphological criteria. Given the taxa we have sampled, our phylogeny suggests that only one proposed subgenus constitutes a natural group, *Putorius*, containing the steppe polecat (*M. eversmanni*), European polecat (*M. putorius*) and the North American black-footed ferret (*M. nigripes*; node 25). Further, *Mustela *is paraphyletic with respect to *Neovison *and suggests that the placement of the American mink in the separate genus *Neovison *may not be warranted despite the observed differences in karyotype and morphology between this taxon and other species of *Mustela*[35]. In the third major clade, grisons of Central and South America (*Galictis*; node 31) are sister to a clade containing the marbled polecat (*Vormela*) of Asia and three African species, the Libyan striped weasel (*Ictonyx libyca*), zorilla (*I. striatus*) and striped weasel (*Poecilogale albinucha*; node 28). Monophyly of these genera is of interest because all exhibit an aposematically colored pelage combined with defense behaviors that include threat displays and excretion of pungent musk from enlarged anal glands [36-39]. Placement of *Vormela *within this clade is congruent with a recent *CYTb *study [40]. Interestingly, there is a north to south progression in branching order from *Vormela *to *I. striatus *and *P. albinucha *of sub-Saharan Africa, which renders *Ictonyx *paraphyletic. This suggests that *P. albinucha *should be placed into the genus *Ictonyx *Kaup, 1835, given the priority of the latter name over *Poecilogale *Thomas, 1883. As for the clade containing *Eira*, *Gulo *and *Martes *(node 34), the last genus is clearly paraphyletic, in agreement with other recent studies [11,13,41,42]. Two New World species, the tayra (*Eira*) and fisher (*Martes pennanti*, subgenus *Pekania*) either comprise a clade or form successive lineages sister to a clade containing wolverine (*Gulo*) and the remaining species of *Martes*. Our phylogeny indicates that true martens (subgenus *Martes*) are monophyletic (node 38) and sister to yellow-throated marten (*M. flavigula*, subgenus *Charronia*). Within the subgenus *Martes*, MP and ML analyses resulted in different phylogenetic placements of *M. americana *and *M. melampus *relative to the (*M. martes *+ *M. zibellina*) clade. In the MP tree, *M. americana *was sister to the clade (*M. melampus *(*M. martes *+ *M. zibellina*)) whereas *M. americana *and *M. melampus *were joined as sister taxa in the ML tree. Relationships of *M. americana*, *M. melampus *and (*M. martes *+ *M. zibellina*) were unresolved in the Bayesian tree, resulting in a trichotomy (Figure 1; node 39). Although more data is required to establish the precise branching order of the fisher, the strong support for this taxon being paraphyletic with the remaining species of *Martes *strongly suggests placement of the fisher in its own genus, *Pekania*.

### Nodal support

Concatenation of 11,929 bp, with indels coded as missing, includes 2,959 (24.8%) variable characters and 1,876 (15.7%) parsimony-informative characters. When indels are coded for information content, the concatenation is reduced to 11,789 bp, with 3,045 (25.8%) variable and 1,917 (16.3%) parsimony-informative characters. The majority of internodes in our phylogeny have >90% bootstrap support (ML and MP) and 1.0 posterior probabilities (BI), see Figure 1 and Table 1, indicating that our phylogenetic hypothesis, given our data set, is robustly supported. Moreover, 14 internodes are supported by one or more synapomorphic indels (Table 1). Three clades, however, have low support in MP, ML and/or BI analyses: nodes 26, 33 and 35. Internodes associated with these clades have very short branch lengths (Figure 1), suggesting that these splits occurred rapidly. Alternatively, speciation may have occurred relatively recently, also resulting in inadequate phylogenetic signal, as among *M. eversmanni*, *M. putorius *and *M. nigripes *(node 26) as well as *M. americana*, *M. melampus *and (*M. martes *+ *M. zibellina*). Many nuclear segments show no differences among species that comprise these groups, suggesting that there has been insufficient time for sequence differences to accumulate at these more slowly evolving loci. Indeed, the four species of *Martes *have been described as a superspecies complex of closely related, yet largely allopatrically distributed taxa [43]. Regardless of the exact cause, short branch lengths of these internodes can result in a high number of anomalous gene trees (and gene tree discordance [44]), leading to an incorrectly inferred species tree, especially when data from multiple data partitions is concatenated [45]. Additional analyses, including population genetic-level sampling, will be needed to confidently resolve relationships among these recently evolved species, as in reference [33] for example.

**Table 1:** Bootstrap values (MP and ML), posterior probabilities (BI), and phylogenetically informative indels that correspond to the 41 nodes shown in Figure 1. Posterior probabilities from BI using two different model-partitioning strategies are shown. Bootstrap values for ML were calculated using a hill-climbing algorithm (ML-hc) and a genetic algorithm (ML-ga); see the methods section. Rows in bold show two nodes (3 and 4) where bootstrap support values were increased when *Arctonyx collaris *and *Meles meles *were excluded from the data set (46 versus 44 taxa) and node 33 corresponds to support values for the sister group relationship between Martinae and the clade (*A. collaris *+ *M. meles*). × = node not present because constituent taxa excluded; ×* = node not recovered in the respective analysis.

Node	MP 46 taxa	MP 44 taxa	ML-hc 46 taxa	ML-hc 44 taxa	ML-ga 46 taxa	BI partitioned	BI uniform	Number of PI indels
1	100	100	100	100	100	1	1	6
2	100	100	100	100	100	1	1	1
3	**71**	**94**	**73**	**95**	82	1	1	
4	**77**	**92**	**90**	**96**	95	1	1	
5	100	100	100	100	100	1	1	
6	82	84	64	63	68	1	1	
7	100	100	100	100	100	1	1	
8	97	98	100	100	100	1	1	
9	100	100	100	100	100	1	1	
10	100	100	100	100	100	1	1	
11	100	100	100	100	100	1	1	1
12	100	100	100	100	100	1	1	
13	100	100	100	100	100	1	1	1
14	100	100	100	100	100	1	1	
15	100	100	100	100	100	1	1	1
16	100	100	100	100	100	1	1	
17	93	95	82	86	86	1	1	1
18	100	100	100	100	100	1	1	1
19	93	93	97	97	98	1	1	
20	78	80	70	71	70	0.9	0.97	
21	100	100	100	100	100	1	1	
22	100	100	100	100	100	1	1	
23	100	100	96	96	100	1	1	1
24	99	99	95	95	99	1	1	
25	99	100	95	95	100	1	1	
26	52	51	59	60	64	1	0.99	
27	100	99	100	100	100	1	1	
28	100	100	100	100	100	1	1	2
29	100	100	100	100	100	1	1	1
30	95	95	100	100	100	1	1	1
31	100	100	100	100	100	1	1	6
32	100	100	100	100	100	1	1	
33	**<50**	**×**	**<50**	**×**	**58**	**0.99**	**0.98**	
34	100	100	100	100	100	1	1	1
35	<50	× *	59	<50	64	0.87	0.87	
36	71	76	92	91	97	1	1	
37	91	92	98	99	99	1	1	
38	100	100	100	100	100	1	1	
39	99	99	100	100	100	1	1	
40	91	93	87	87	76	0.87	1	
41	100	×	100	×	100s	1	1	3

Even so, internodes with low support may be stable or unstable, depending on the relative stability or instability of their constituent (terminal) taxa or leaves [46]. Further, unstable taxa can influence nodal support in other parts of a phylogenetic tree [47]. We measured leaf stability of all taxa using bootstrap trees from MP analyses and the program RadCon [48]. For three different measures of leaf stability, *Arctonyx collaris *and *Meles meles *were the least stable taxa (see Additional file 1). We repeated MP and hill-climbing ML analyses (see the methods section) along with bootstrapping after excluding these two taxa, thereby reducing the data set to 44 taxa. Most bootstrap values in these analyses showed little or no change compared with the 46 taxa data set, but interestingly, support for two stem clades (nodes 3 and 4) increased from over 70% to more than 90%, as did average phylogenetic stability (Table 1). The lower stability of *A. collaris *and *M. meles *and their influence on bootstrap support at internodes 3, 4 and 33 may be caused by character conflict among different gene segments associated with these two taxa [49]. However, we note that these nodes all have high or maximal posterior probabilities in BI analyses (Table 1).

With regards to subfamilial classification, our phylogeny clearly indicates that the Mustelinae, as both traditionally [5] and recently [7] conceived, is polyphyletic, as suggested by previous studies [8,13,14]. Our results also indicate that various genera of badgers (*Arctonyx*, *Meles*, *Mellivora*, *Melogale *and *Taxidea*), most of which are often placed in the Melinae [5], are also polyphyletic, supporting earlier conclusions based on morphology that these taxa are not closely related [8,50,51]. Instead, badgers with specific adaptations for fossoriality (*Arctonyx*, *Meles*, *Mellivora *and *Taxidea*) constitute a basal grade of lineages that are best recognized as distinct subfamilies. Overall, our phylogeny (Figure 1) is consistent with the subfamilial classification scheme recently proposed by Sato et al [15] and Fulton and Strobeck [11], which redefines the boundaries of traditional subfamilies (e.g. Mustelinae) as well as resurrects other subfamilies such as Helictidinae and Mellivorinae, thereby reflecting actual phyletic lines that have been difficult to resolve based on morphology alone.

### Divergence times

Divergence times across the mustelid phylogeny were estimated using the uncorrelated relaxed lognormal molecular clock model calibrated simultaneously by eight fossil constraints. Use of this molecular clock model provides a measure of rate heterogeneity among lineages or how well data conform or deviate from a strict molecular clock [52]. For the root age and crown age prior combination of 28.5 MYA and 24 MYA, the coefficient of variation (σ_r_) averaged across three independent runs was 0.375 (95% highest posterior density (HPD): 0.274–0.486), which suggests that the data show rate heterogeneity among lineages (i.e. the concatenated data are evolving in a non-clocklike manner). We found greater rate heterogeneity among lineages when older root age and crown age prior combinations were used; for example, for 33.7 MYA and 28.5 MYA, averaged across three runs, σ_r _= 0.504 (95% HPD: 0.381–0.631). Furthermore, a likelihood ratio test significantly rejects the ML phylogeny with an enforced molecular clock versus one without a molecular clock (-ln *L*_with clock _= 55179.13 and -ln *L*_without clock _= 55095.88; *p *< 0.001) thereby providing additional support that our data departs from clocklike behavior.

In general, mean posterior estimates as well as 95% HPD intervals of node ages were highly consistent among different combinations of root age and crown age prior combinations (Table 2). The root and node 1 (the divergence between *Taxidea *and remaining mustelids) were most sensitive to alterations in priors, because they showed the largest shift in divergence times (~5 million years) and little overlap in 95% HPD intervals in runs using older versus younger combinations of root age/crown age priors (Table 2). Nonetheless, sampling of the joint prior distribution by performing Markov chain Monte Carlo (MCMC) analyses without any data (using BEAST v1.4.2 [52]) suggested that the eight fossil calibration point priors (as well as the various root age and crown age combinations) did not have a strong influence on their estimated posterior distributions (see Additional file 2) and thus, estimated divergence times.

**Table 2:** Estimated divergence times derived from Bayesian relaxed molecular clock analyses using a combination of root age priors and minimum crown age priors. Mean and 95% HPD of the posterior probability distribution are in MYA. Node numbers correspond to those shown in Figure 2 (same as in Figure 1).

	**24 MYA root prior**	**28.5 MYA root prior**		**33.7 MYA root prior**	
	
	**24 MYA**	**24 MYA**	**28.5 MYA**	**24 MYA**	**28.5 MYA**
	
**Node**	**Mean [95% HPD]**	**Mean [95% HPD]**	**Mean [95% HPD]**	**Mean [95% HPD]**	**Mean [95% HPD]**
Root	24.2 [22.3–26.0]	28.5 [26.6–30.4]	28.6 [26.7–30.4]	33.6 [31.6–35.5]	33.7 [31.7–35.6]
1	20.9 [18.8–22.9]	21.0 [19.0–23.1]	26.1 [24.1–28.1]	21.2 [19.3–23.2]	26.2 [24.1–28.2]
2	12.4 [11.0–13.7]	12.5 [11.2–13.9]	12.6 [10.9–14.2]	12.5 [11.1–13.9]	12.6 [10.9–14.1]
3	11.6 [10.1–13.0]	11.8 [10.4–13.1]	11.9 [10.3–13.7]	11.8 [10.4–13.2]	11.9 [10.2–13.5]
4	10.8 [9.4–12.2]	11.0 [9.7–12.4]	11.1 [9.3–12.7]	11.0 [9.5–12.5]	11.0 [9.3–12.8]
5	9.1 [7.7–10.4]	9.2 [7.8–10.5]	9.5 [7.9–11.0]	9.3 [7.8–10.7]	9.3 [7.6–10.9]
6	8.7 [7.3–10.0]	8.8 [7.5–10.2]	9.0 [7.3–10.6]	8.9 [7.5–10.3]	8.8 [7.3–10.4]
7	7.4 [6.0–8.9]	7.6 [6.2–9.0]	7.7 [6.0–9.6]	7.6 [6.1–9.0]	7.6 [6.0–9.3]
8	6.4 [4.9–7.8]	6.5 [5.1–7.9]	6.5 [4.7–8.3]	6.6 [5.1–7.9]	6.5 [4.9–8.2]
9	4.9 [3.6–6.1]	4.9 [3.7–6.1]	4.8 [3.5–6.2]	5.0 [3.9–6.3]	5.0 [3.5–6.5]
10	3.6 [2.7–4.6]	3.7 [2.8–4.6]	3.6 [2.5–4.8]	3.7 [2.9–4.8]	3.7 [2.6–4.8]
11	2.6 [1.8–3.5]	2.7 [1.8–3.5]	2.4 [1.4–3.4]	2.6 [1.8–3.6]	2.5 [1.6–3.4]
12	1.4 [0.8–2.2]	1.4 [0.8–2.1]	1.3 [0.6–2.1]	1.4 [0.8–2.1]	1.4 [0.7–2.1]
13	1.8 [0.9–2.7]	1.8 [1.0–2.8]	1.8 [0.7–2.9]	1.8 [0.9–2.7]	1.8 [0.6–3.1]
14	2.8 [1.7–4.0]	2.8 [1.9–4.0]	3.1 [1.6–4.7]	2.8 [1.8–4.1]	3.4 [1.7–5.2]
15	1.5 [0.7–2.4]	1.5 [0.7–2.3]	1.6 [0.5–2.7]	1.5 [0.7–2.3]	1.8 [0.6–3.2]
16	6.1 [4.9–7.2]	6.2 [5.1–7.3]	6.1 [4.8–7.3]	6.1 [5.0–7.2]	6.0 [4.6–7.3]
17	5.2 [4.1–6.4]	5.3 [4.2–6.3]	5.1 [3.8–6.4]	5.2 [4.2–6.3]	5.0 [3.6–6.2]
18	3.5 [2.7–4.3]	3.6 [2.8–4.4]	3.5 [2.6–4.4]	3.6 [2.8–4.4]	3.5 [2.4–4.5]
19	2.8 [2.1–3.5]	2.8 [2.1–3.6]	2.8 [1.9–3.6]	2.8 [2.1–3.5]	2.8 [1.9–3.6]
20	2.2 [1.5–3.0]	2.3 [1.5–3.0]	2.1 [1.1–3.1]	2.2 [1.5–3.0]	2.1 [1.1–3.0]
21	3.2 [1.7–4.6]	3.3 [1.9–4.8]	3.1 [1.5–4.7]	3.3 [1.8–4.8]	3.3 [1.6–5.1]
22	2.9 [1.6–4.1]	2.9 [1.8–4.1]	2.8 [1.2–4.3]	2.9 [1.8–4.0]	2.8 [1.4–4.3]
23	1.6 [1.1–2.2]	1.6 [1.1–2.2]	1.8 [1.1–2.6]	1.7 [1.1–2.2]	1.8 [1.0–2.5]
24	1.2 [0.7–1.6]	1.2 [0.4–1.6]	1.3 [0.7–1.9]	1.2 [0.8–1.7]	1.3 [0.7–1.9]
25	0.6 [0.3–0.9]	0.6 [0.4–0.9]	0.7 [0.3–1.1]	0.6 [0.4–0.9]	0.7 [0.3–1.1]
26	0.4 [0.2–0.7]	0.4 [0.2–0.7]	0.4 [0.1–0.8]	0.4 [0.2–0.7]	0.4 [0.1–0.8]
27	7.9 [6.3–9.6]	8.1 [6.5–9.6]	8.1 [6.1–10.0]	8.2 [6.6–9.8]	7.9 [5.8–10.1]
28	4.5 [3.3–5.8]	4.6 [3.5–5.8]	4.0 [2.8–5.2]	4.6 [3.4–6.0]	4.0 [2.6–5.3]
29	3.4 [2.4–4.4]	3.5 [2.5–4.5]	3.0 [2.1–4.1]	3.5 [2.3–4.6]	3.0 [1.8–4.3]
30	2.6 [1.6–3.6]	2.6 [1.7–3.6]	2.2 [1.1–3.2]	2.7 [1.5–3.7]	2.2 [1.1–3.3]
31	2.8 [1.5–4.3]	2.8 [1.4–4.3]	2.9 [1.1–5.0]	3.0 [1.6–4.6]	2.8 [1.1–5.1]
32	2.2 [0.9–3.7]	2.3 [1.0–3.6]	2.4 [0.7–4.3]	2.2 [0.9–3.5]	2.5 [0.9–4.5]
33	11.0 [9.4–12.5]	11.1 [9.7–12.6]	11.1 [9.3–12.9]	11.1 [9.6–12.8]	11.1 [9.2–12.9]
34	6.8 [5.1–8.5]	6.9 [5.2–8.7]	7.7 [5.4–10.0]	6.7 [4.7–8.5]	7.1 [5.0–9.3]
35	6.4 [4.7–8.0]	6.5 [4.9–8.2]	7.2 [5.0–9.5]	6.3 [4.5–8.1]	6.6 [4.8–8.8]
36	5.6 [4.0–7.1]	5.7 [4.1–7.3]	6.2 [4.0–8.3]	5.5 [3.7–7.3]	5.8 [4.0–7.8]
37	4.8 [3.4–6.3]	4.8 [3.4–6.4]	5.1 [3.1–7.4]	4.7 [3.0–6.4]	4.8 [3.1–6.7]
38	2.8 [1.9–3.7]	2.8 [1.9–3.8]	3.0 [1.8–4.2]	2.8 [1.9–3.7]	3.1 [2.0–4.2]
39	1.6 [1.1–2.2]	1.6 [1.0–2.3]	1.7 [1.0–2.6]	1.6 [1.0–2.2]	1.8 [1.1–2.6]
40	1.0 [0.5–1.6]	1.1 [0.6–1.6]	1.1 [0.4–1.8]	1.0 [0.5–1.6]	1.5 [0.5–2.0]
41	3.6 [1.8–5.9]	3.7 [1.8–6.0]	4.4 [1.5–7.1]	3.7 [1.9–5.7]	4.2 [1.6–7.2]

**Figure 2:**
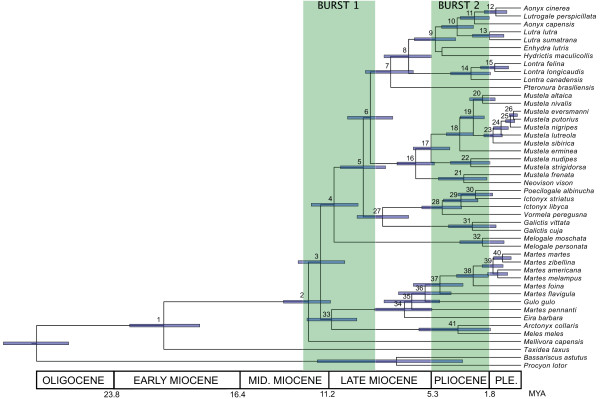
**Chronogram of the Mustelidae based on Bayesian analysis**. Posterior values of branch lengths and divergence times (in millions of years) were estimated using 28.5 MYA as the root age prior and 24 MYA as the minimum age for the crown group, the GTR + I + G model of DNA substitution and the uncorrelated lognormal relaxed molecular clock model (rate of each branch is sampled independently from a lognormal distribution, with rates assumed to be uncorrelated among branches). Nodes are numbered as in Figure 1 and posterior estimates of mean and 95% HPD of divergence times are presented in Table 2. Bars represent 95% HPD around mean estimates of divergence times. Vertical green bars indicate two bursts of diversification. Geological time scale is shown at bottom.

How do our estimates of divergence times compare with those based on previous molecular studies of mustelids [12,14,17,41]? At nodes shared among the different studies, divergence times in previous studies are either a mix of younger, older and overlapping dates relative to those in our study [17] or they tend to be older in general [12,14,41]. For example, the estimated divergence time for the split between *Neovison vison *and the remaining taxa of *Mustela *was dated at 8.5–9.9 MYA, 6.6–9.5 MYA and 10–14 MYA in Sato et al [14], Marmi et al [17] and Hosoda et al [41], respectively. In our study, this split (node 16; Figure 2 and Table 2), which also includes *M. frenata *as sister to *N. vison*, is dated at ~6.0 MYA (95% HPD: 4.6–7.3 MYA). However, earlier studies differ in several important respects from our study. First, these studies were based on a smaller number of taxa and loci. For example, in Sato et al [14] and Hosoda et al [41], taxon sampling largely consisted of species from *Martes *and *Mustela *and was based on one and two loci, respectively. Undersampling of taxa and characters can bias divergence time estimates [53-55] and reduces the power to detect rate variation among lineages [56]. Second, previous studies all employed a single root age fossil constraint, treated as a hard bound, precluding an accurate estimation of error associated with this calibration point. In contrast, we employed multiple fossil constraints simultaneously and placed soft-bound priors on these constraints to account for the uncertainty associated with the fossil record, thereby making it possible to evaluate errors associated with divergence time estimates [57,58]. Third, the previous studies each estimated a substitution rate that was assumed to be constant across their respective phylogenies. However, our results indicate that substitution rates vary across different lineages of the mustelid phylogeny (see above), consistent with recent evidence from different groups that rates of substitution vary across lineages, even among closely related species [59].

### Tempo and mode of mustelid diversification

**Figure 3:**
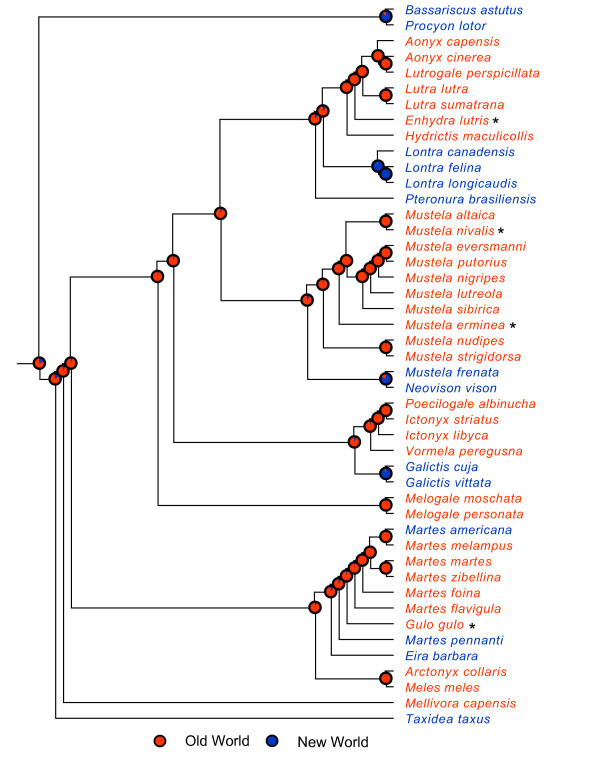
**ML phylogeny of Mustelidae showing reconstruction of ancestral areas based on the two-state analysis**. Pie charts at nodes show proportional likelihoods that the common ancestor was distributed in the Old World (blue) or New World (red). Proportional likelihood values and associated significance levels for ancestral area reconstructions are presented in Additional file 3. Taxa are colored according to their coding states (see legend). * = occurs in both Old and New World.

**Figure 4:**
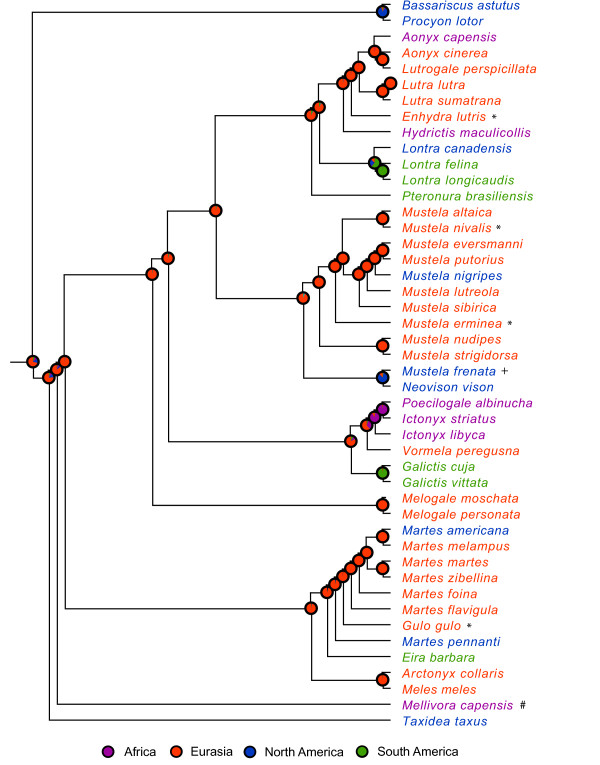
**ML phylogeny of Mustelidae showing reconstruction of ancestral areas based on the four-state analysis**. Pie charts at nodes show proportional likelihoods that the common ancestor was distributed in Africa (purple), Eurasia (red), North America (blue) or South America (green). Proportional likelihood values and associated significance levels for ancestral area reconstructions are presented in Additional file 3. Taxa are colored according to their coding states (see legend). * = occurs in Eurasia and North America; + = occurs in North America and South America; # = occurs in Africa and Eurasia.

Using the root age and crown age prior combination of 28.5 MYA and 24 MYA as a reference, our dating analyses indicate that, following the initial divergence of *Taxidea *in the Early Miocene (21.0 MYA, 95% HPD: 19.0–23.1 MYA), mustelids underwent two main bursts of diversification (Figure 2). The first burst occurred during a ~3.7 million year interval from the Middle to Late Miocene (12.5-8.8 MYA, 95% HPD: 13.9-7.5 MYA) and gave rise to most of the extant primary clades and lineages (nodes 2–6 and 33). The second and larger burst occurred during the Pliocene epoch (5.3-1.8 MYA) in which as many as 20 generic-level or specific-level lineages originated within a 3.5 million year span of time (Figure 2 and Table 2). Furthermore, results of our biogeographic analyses show that the majority of cladogenetic events associated with these bursts of diversification occurred in the Old World, largely in Eurasia (Figures 3 and 4; see below).

Paleoenvironmental and biotic changes driven by changes in climate during the latter half of the Neogene may have promoted the two bursts of cladogenesis within mustelids. Following the Mid-Miocene Climatic Optimum and onset of modern oceanic circulation (17-15 MYA), there is a marked cooling of the global climate near the end of the Middle Miocene that continues through to the Holocene [60]. This period of cooling coincides with formation of a permanent Antarctic ice sheet in the Mid to Late Miocene and an Arctic ice sheet in the Pliocene [60]. In addition, several major sea-level lowstands occurred during the Late Miocene and Pliocene, including the Serravallian sea-lowering event near the beginning of the Late Miocene, 11-10 MYA [61,62]. These changes in climate and sea level increased overall terrestrial aridity and seasonality, which in turn promoted a shift from closed vegetation habitats (tropical and subtropical forests) to more open vegetation habitats (woodlands and grasslands) [63-65]. By the early Late Miocene, plant and animal fossil evidence indicates that the Eurasian continent was a mosaic of vegetation types and generally more heterogeneous in vegetation structure relative to that in the Early to Middle Miocene [66]. These changes in vegetation had a concomitant impact on faunal communities and may have fostered diversification in a variety of lineages, including mustelids, via geographic isolation, divergent selection among different habitats, and/or ecological opportunity through the creation of new niches or the reorganization of former niches. Interestingly, the initial radiation of the primary clades and lineages of mustelids coincides with a major faunal turnover in Western Europe (the middle Vallesian 'crisis') that affected many groups of mammals, including the Carnivora [62,67]. In fact, nearly half of carnivoran species that went extinct during this turnover were mustelids [68] and turnover in mustelids remained high throughout the Late Miocene in western Eurasia [69]. Although evidence for faunal change in other parts of Eurasia is less clear, changes in habitat and extinction of earlier lineages of mustelids may have created ecological opportunities that fostered the initial burst of diversification of modern mustelids.

Further cooling and drying during the Pliocene, coincident with onset of high latitude glacial cycles [60], caused a dramatic expansion of low-biomass vegetation, including grasslands and steppe at midlatitudes and development of taiga at high latitudes of Eurasia and North America [63-65]. Coupled with these changes was diversification of prey species such as rodents (particularly muroid rodents) and passerine birds that exploited these new habitats, which in turn provided new niches for predators [68,70]. Part of the Pliocene burst of diversification of mustelids may have been promoted by this diversification of prey species, as species in genera such *Martes *(nodes 37–39 in Figure 2) and especially *Mustela *(nodes 17–23 in Figure 2) became specialized in hunting small prey such as rodents. Such a scenario is consistent with King's [71] hypothesis that evolution of small body size in *Mustela *was partly driven by adaptation to exploit abundant resources presented by rodent diversification during the Pliocene. We also note that diversification of four of the five species within the subgenus *Martes *(*M. americana*, *M. martes*, *M. melampus *and *M. zibellina*; node 39), which are all closely associated with taiga forest habitat [72], coincides with expansion of this type of habitat across the Holarctic during the Plio-Pleistocene [63]. This finding is consistent with fossil evidence that indicates that taxa ancestral to these living species primarily evolved in forested habitats [43,73]. In Africa, the split between *Ictonyx libyca *and the clade containing *I. striatus *and *Poecilogale albinucha *(node 29) dated at 3.0–3.5 MYA (95% HPD: 1.8–4.6 MYA), marginally overlaps with a major increase in African aridity and climate variability that occurred 2.9-2.4 MYA, according to paleoclimatic and faunal evidence [74,75]. Moreover, this interval was accompanied by rapid radiation in several mammalian lineages such as bovids and hominids [74,75]. Our results suggest that divergence of *I. libyca*, which occurs in North Africa along margins of the Sahara desert, and the clade containing *I. striatus *and *P. albinucha*, which both occur south of the Sahara, may have also been caused by this shift to greater aridity, especially considering that extensive desert conditions in the Sahara did not occur until the Late Pliocene, around 2.8 MYA [74,76,77].

**Figure 5:**
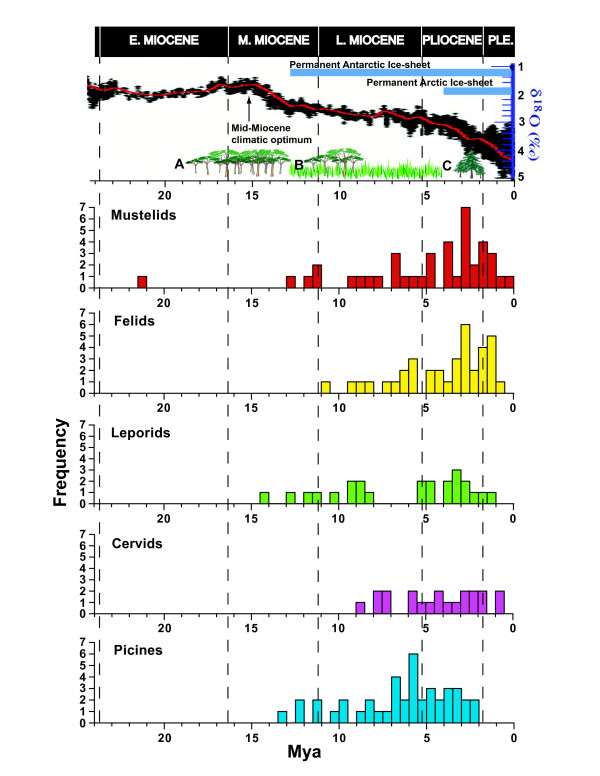
**Frequency histograms showing distribution of node ages within mustelids and four other vertebrate taxa**. Mean node ages for mustelids are based on 28.5 MYA root age and 24 MYA crown age priors (see Table 2). The top panel shows ocean temperature curve (smoothed mean in red) based on global deep-sea oxygen isotope (δO^18^) records (modified from [60]). The development of full-scale ice-sheets in each hemisphere as well as key changes in vegetation in the northern hemisphere during the Neogene are also shown. Following the Mid-Miocene climatic optimum, forested habitats (A) gave way to more open vegetation habitats such as woodlands and grasslands (B). Taiga forests greatly expand during the Pliocene (C). See the text for further details. PLE. denotes Pleistocene.

If mustelid diversification was promoted by climatically driven paleoenvironmental changes, then synchronous patterns and tempos of diversification should also be recorded in the evolutionary histories of other, unrelated groups. A number of recent molecular phylogenetic studies on various groups of mammals and other vertebrates employing multiple loci and relaxed molecular clock methods provide corroboration for this hypothesis. For example, squirrels (Sciuridae [32]), cats (Felidae [78]), rabbits and hares (Leporidae [79]), deer (Cervidae [80]) and woodpeckers (Picinae [81]) each show one or more episodes of rapid diversification that are roughly contemporaneous with one or both of those observed in mustelids (Figure 5). Taking cats as an example, seven of the eight primary lineages of felids radiated in the early part of the Late Miocene (10.8-6.2 MYA) whereas genera and species that comprise the eight lineages largely radiated during the Pliocene [78]. We also note that divergence events from a number of independent lineages within the Mustelidae are synchronous as well. For example, using the root age and crown age prior combination of 28.5 MYA and 24 MYA as reference, nodes 11, 14, 19, 22, 31 and 38 all codiversify around 2.8 MYA (Figure 2 and Table 2). Similarly, nodes 9, 28 and 37 overlap in their divergence time estimates of around 4.8 MYA (Table 2). While ecological circumstances obviously differ from group to group, such congruence in patterns and tempos of diversification supports the idea that a common cause, namely, large-scale changes in past environments, has shaped the phylogenetic histories among disparate groups of organisms as well as independent lineages of mustelids.

Our results have important implications for theories about the mode and tempo of adaptive radiations. First, the scenario of diversification across different trophic groups we have outlined above is consistent with the model of cascading radiation proposed by Stanley [82], in which diversification of one group in a trophic cascade (e.g. producers such as grasses and taiga) promotes diversification in a second group (e.g. primary consumers such as rodents), which in turn promotes diversification in a third group (secondary consumers such as mustelids). This model could be tested more rigorously using dated phylogenies of multiple groups representing different trophic levels. Second, our finding that the two primary bursts of cladogenesis in the evolutionary history of extant mustelids (and perhaps in the other groups cited above) coincided with periods of climatically mediated environmental changes provides support for models that show that evolution may be greatly accelerated in temporally and spatially changing environments [83]. During these times, new niches are created or former niches are reorganized, providing new ecological opportunities that may foster rapid speciation and thus, diversification [2,84] (see also [85]). The fact that multiple groups show contemporaneous periods of rapid cladogenesis (e.g. mustelids, felids and leporids during the Pliocene) suggests that mainland environments undergoing environmental changes may function like newly colonized island archipelagoes in promoting diversification.

### Historical biogeography

The two-state and four-state biogeographical reconstructions indicate that the vast majority of the modern diversification of mustelids has occurred in the Old World (Figure 3), specifically in Eurasia (Figure 4). In fact, Eurasia was unambiguously reconstructed as the ancestral area for nearly every ancestral node in the four-state analysis (Figure 4). These results are consistent with two other observations that suggest Eurasia has been the center of mustelid diversification: (1) Eurasia contains the majority of extant species, with 34 of the 59 known species either exclusively endemic to or having part of their distribution on this continent; and (2) the earliest fossils of extant lineages or those associated with the ancestors of extant lineages are often found in Eurasia [23]. Nodes located near the base of the tree tend to be those where likelihood ratios are not significant (and, thus, ancestral reconstruction is inferred as equivocal), such as the root node and the node joining *Taxidea *as sister to the remaining taxa of mustelids in the two-state analysis (see Figure 3 and Additional file 3). Such results are not uncommon in likelihood or other model-based approaches to ancestral state reconstruction because the degree of uncertainty associated with reconstruction increases with time [86]. Nonetheless, proportional likelihood values for these nodes still favor the Old World as the ancestral area in the two-state analysis (see Figure 3 and Additional file 3). Ancestral reconstructions were robust when *Enhydra lutris*, *Gulo gulo *and *Mellivora capensis *were coded for alternative states (results not shown; see methods).

In contrast to the extensive *in situ *diversification that has taken place in Eurasia, mustelid faunas of Africa and the New World are largely comprised of genera or species that repeatedly colonized these regions from Eurasia. The mustelid fauna of Africa contains eight species, seven of which are included in our taxon sampling (the Egyptian weasel, *Mustela subpalmata*, was not sampled). Of these seven species, five are derived from separate colonizations from Eurasia (*Aonyx capensis*, *Hydrictis maculicollis*, *Mustela putorius*, the *Ictonyx *+ *Poecilogale *lineage and *Mellivora capensis*) whereas two are derived from *in situ *speciation events (*Ictonyx striatus *and *P. albinucha*); see Figure 4. Similarly, our reconstructions show that nine separate dispersal events from Eurasia and only one *in situ *speciation event accounts for the diversity of mustelids that are either endemic to North America (e.g. *Lontra canadensis*, *M. nigripes*, *Martes pennanti*) or have part of their distribution there (e.g. Holarctic species such as *Gulo gulo*, *Mustela erminea *and *M. nivalis*). Genera and species of mustelids found in South America today are largely descended from North American immigrants that arrived as part of the Great American Interchange following the rise of the Panamanian isthmus, 3.0-2.5 MYA [23,87,88]. Such a relationship is clearly indicated for the clade of New World otters in which *L. canadensis *is sister to *L. felina *+ *L. longicaudis*, with the latter two species found in Central and/or South America (Figure 4). Moreover, this split (node 14) is estimated to have occurred 2.8–3.4 MYA (95% HPD: 1.6–5.2 MYA), which overlaps well with timing of the formation of the Panamanian land bridge. The long-tailed weasel, *M. frenata*, ranges from North America to northern South America [89] and two species of weasels (*M. africana *and *M. felipei*, not sampled here) are endemic to South America. Fossil evidence clearly indicates that *Mustela *colonized South America from the north, apparently well after the Panamanian isthmus was in place [23,90]. Our results show that *Pteronura*, *Galictis *and *Eira *dispersed separately into South America, with Eurasia reconstructed as the continent of origin for each genus (Figure 4). These results are anomalous because these genera have been allied with extinct taxa from North America, suggesting a more proximate origin for these lineages [91,92]. For example, *Pteronura *may be related to the extinct genus *Satherium *from the Pliocene of North America [91]. However, paleontological studies suggest that the ultimate ancestry of these extinct taxa lies in Eurasia [92,93].

A combination of evidence from the fossil record and divergence times from our phylogeny indicates that the mustelid faunas of Africa, North America and South America have been assembled gradually over time. For example, fossil evidence suggests mustelids colonized the New World across Beringia during different intervals when the land bridge between Eurasia and North America was open. Multiple genera of mustelids entered North America during the Late Miocene (~11.2-5.3 MYA [19,23]), prior to the first opening of the Bering Strait 5.4–5.5 MYA, which severed the route across Beringia [94,95]. Many genera that colonized North America during the Late Miocene or earliest Pliocene became extinct [19,20]. Nonetheless, among the genera that may have been included in this wave of dispersal were the earliest representatives of *Lutra *(which may represent *Lontra*, given that New World river otters have been reclassified into *Lontra*) and *Mustela*, both of which are first recorded in North America from the Late Miocene to Early Pliocene (~5.9-4.6 MYA [19]). These taxa may have been the forerunners of modern species of *Lontra *and *Mustela*/*Neovison *found in North and South America today. The Late Miocene divergence time of the splits leading to *Lontra *and *Neovison *+ *M. frenata *(nodes 8 and 16 in Figure 2) are consistent with this possibility. Two extinct genera of American badgers, *Chamitataxus *and *Pliotaxidea*, are recorded from the Late Miocene, around 7.3 MYA and 6.5 MYA, respectively [96,97]. *Pliotaxidea *has been shown to be sister group to *Taxidea *based on morphological evidence [98], thereby suggesting that the lineage leading to *Taxidea *arrived in North America before the opening of the Bering Strait. Meline badgers (*Arctonyx *and *Meles*) are presently found only in the Old World. However, recent discovery of Late Miocene to Early Pliocene fossils of meline badgers at two different sites in North America [99,100] indicates that this lineage had also immigrated into North America and was a component of the New World mustelid fauna.

Following these earlier dispersal events, fossil evidence indicates that *Mustela erminea*, *M. nigripes*, *M. nivalis *and *Martes americana *later entered North America during the Pleistocene [26,102]. The molecular divergence time for *M. nigripes *(node 25), around 0.6 MYA (95% HPD: 0.3–1.1 MYA; Table 2), for example, supports a Pleistocene dispersal scenario for this species. Although fossil records of mustelids in Africa and South America are less well known than those of the northern continents, first appearance datums of both extant and extinct genera of mustelids nevertheless suggest that these regions also were colonized through successive dispersal events [23,102-105]. Among the extinct genera known from Late Miocene deposits in East Africa that may have arisen from Eurasian immigrants are the gigantic and cat-like *Ekorus*, the largest mustelid discovered thus far, and an otter, *Vishnuonyx *[105]. In contrast, the earliest known remains of *Ictonyx striatus *are from the Pleistocene [106], suggesting a later immigration into Africa.

### Implications for mustelid community ecology

Most studies of modern-day mustelid communities (where multiple species coexist in a single area of a geographic region) have focused on recent ecological factors, such as partitioning of resources (food or space) via competition, to explain coexistence of species within these communities [29,107-109]. As these studies have operated on an ecological timeframe, they implicitly assume that ecological differences among species have evolved relatively recently. However, several observations derived from our biogeographical and phylogenetic results suggest that history (via phylogeny) also has been an important component in the structure of mustelid assemblages on different continents.

First, these assemblages are largely composed of species belonging to different clades or lineages that differ significantly in their ecomorphology with regards to diet and locomotor mode [110,111]. For example, seven species of mustelids are sympatric on the British Isles in Eurasia, with three species of weasels, (*Mustela*) and one species each of mink (*Neovison*, introduced), marten (*Martes*), badger (*Meles*) and otter (*Lutra*) [29]. Weasels, martens, badgers and otters obviously comprise a phylogenetically and ecologically heterogeneous set of species, yet these fundamental differences may indeed facilitate coexistence of these species within this community. Even the three species of weasels found in this community (*M. erminea, M. nivalis *and *M. putorius*) are not closely related (Figure 1), suggesting that resource partitioning among these species may also be in part determined by historical causes.

Second, studies of other vertebrate communities (e.g. lizards) have demonstrated that competition and divergence in ecological traits varies according to the degree of phylogenetic relatedness among species, such that, for example, competition is expected to be low among distantly related species but high among closely related species [112,113]. Divergence in ecological traits should therefore be greatest between closely related species, especially sister species occupying the same area. Within mustelids, there are several instances where sister taxa that are broadly sympatric within a region often exhibit pronounced ecological differences. In Africa, *Poecilogale albinucha *preys almost exclusively on rodents whereas *Ictonyx striatus *has a more generalized diet that includes invertebrate prey [36,37]. Similarly, *Mustela frenata *and *Neovison vison *overlap extensively in North America, but differ significantly in several aspects of their ecologies [89,114]. The sister taxa *Aonyx cinerea *and *Lutrogale perspicillata *co-occur in parts of south and southeast Eurasia with the former species feeding largely on freshwater crabs and the latter feeding mostly on fish [115,116].

Third, the fossil record along with molecular dating results indicate that continental assemblages of mustelids have been built up gradually over time (see the above discussion). This suggests that species interactions with these communities are temporally mosaic, with some interactions being ancient while others are more recent. We certainly do not suggest that present-day ecological factors (e.g. interspecific competition for food and/or space, predation) operating over ecological time scales have not been important in determining the structure of modern-day mustelid communities. Instead, we simply suggest that historical factors should also be considered and that such a perspective has been lacking in most previous studies of mustelid community ecology. As other researchers have pointed out, both historical factors and more recent ecological processes contribute to the structure of modern day communities [113,117].

## Conclusion

We have reconstructed a nearly complete generic-level phylogeny of the Mustelidae based on a supermatrix of 22 gene segments. Using a variety of phylogenetic reconstruction methods, we have shown that mustelids are consistently resolved into four primary clades and three monotypic lineages and that nearly all nodes for this topology are well supported. Furthermore, by applying Bayesian dating techniques we have shown two bursts of diversification, first during the Miocene, which gave rise to the primary extant clades and lineages, and another during the Pliocene, which gave rise to a large proportion of the species diversity observed today. These bursts of diversification coincided with major paleoenvironmental and biotic changes that occurred during the Neogene and are broadly contemporaneous with periods of rapid cladogenesis in other vertebrate groups. Such concordance in pattern and tempo of diversification suggests that global-scale changes have shaped the histories of many diverse groups of taxa [32]. We used ancestral reconstruction of biogeographic areas to show that most of the extant diversity of mustelids originated in Eurasia. Further, the mustelid fauna of Africa, North America and South America are composed of taxa from nearly all major clades and lineages, suggesting that *in situ *speciation has been a relatively minor component in the assembly of these faunas. Finally, divergence times estimated from the molecular data combined with the fossil record suggests that different lineages of mustelids dispersed to Africa, North America and South America in successive waves, which has implications for understanding the structure of mustelid communities.

## Methods

### Taxon sampling

We obtained tissue samples from mustelid taxa representing 21 of 22 putative genera and 43 of 58 extant putative species, following the classification of Wozencraft [7]; (see Additional file 4). Samples were unavailable from *Lyncodon patagonicus*, seven species of *Mustela*, two species of *Meles*, two species of *Melogale *and one species each of *Lontra*, *Lutra *and *Martes*. Multiple lines of evidence indicate that Procyonidae is the sister group of Mustelidae [10,16]. Therefore, two species of Procyonidae, *Procyon lotor *and *Bassariscus astutus*, were used as outgroups to root the mustelid tree.

### Sequence data collection

Total genomic DNA was extracted from hair or tissue samples using phenol chloroform, followed by ethanol precipitation [118] or using a QIAamp DNA Mini Kit (Qiagen, Valencia, CA). Twenty-one nuclear gene segments were amplified using published primers (see Additional file 5) and the complete mitochondrial cytochrome *b *(*CYTb*) gene was amplified with primers as described in Koepfli and Wayne [13]. Polymerase chain reaction (PCR) was carried out in MWG-Biotech Primus 96 Plus thermal cyclers with the following conditions: 28–30 cycles of 94°C for 30 s; 50–56°C for 30 s; 72°C for 45 s; and one cycle of 72°C for 5 min. Each 50 μl reaction contained 35.7 μl sterile double-distilled water, 5 μl 10× PCR buffer, 5 μl of 25 mM MgCl_2_, 1 μl of 10 mM dNTP mix, 1 μl of both 25 pM/μl forward and reverse primers, 0.3 μl *Taq *polymerase (Sigma-Aldrich, St Louis, MO) and 1 μl of 0.1–1 μg genomic DNA. All PCRs included a negative control (no DNA). PCR products were electrophoresed in and excised from 1% agarose/Tris-acetic acid-EDTA gels and purified using an Ultra Clean Kit (MoBio Laboratories, Solana Beach, CA). PCR products were then cycle sequenced using the original amplification primers and either the CEQ Dye Terminator Cycle Sequencing Quick Start Kit (Beckman Coulter, Fullerton, CA) or BigDye Terminator v3.1 Cycle Sequencing Kit (Applied Biosystems, Foster City, CA). Sequencing reactions were run through either a CEQ 2000 XL DNA Analysis System or an Applied Biosystems 3730 DNA Analyzer. Sequence chromatograms were checked for accuracy and edited using Sequencher 3.1 (Gene Codes Corporation, Ann Arbor, MI).

For several species, DNA extracts from hair samples did not yield a sufficient amount of DNA for direct amplification of nuclear gene segments. We therefore first whole-genome amplified these samples using the GenomiPhi V2 DNA Amplification Kit (Amersham Biosciences, Little Chalfont, UK) and then proceeded with regular PCR protocols (as above). To ensure that the whole-genome amplification process had not introduced any errors into our target sequences, we amplified the whole genome from the same sample a second time and then amplified and sequenced several nuclear loci to compare sequences from the two samples (all were identical).

*Mustela altaica *was represented by only three of the 22 gene segments: *CYTb*, *RAG1 *and *APOB *(exon 26). Sequences for these segments were downloaded from Genbank and were from studies by Sato et al [15,16] and Kurose et al [33], thereby increasing the ingroup to 44 taxa. *RAG1 *sequences for *Gulo gulo*, *Martes flavigula*, *M. foina*, *M. martes*, *M. zibellina*, *Melogale moschata*, *Mustela altaica *and *M. erminea *were downloaded from Genbank from the study by Sato et al [15]. All new sequences were deposited in Genbank and accession numbers for all sequences are presented in Additional file 6.

We were unable to obtain sequences for one or more gene segments from nine of the 46 species sampled. Species and the segments they are missing are: *Galictis cuja *(*BRCA1 *[fragment 2]); *Ictonyx libyca *(*ATP7A*); *Martes zibellina *(*PLCB4*); *Mellivora capensis *(*ADORA3*); *Melogale personata *(*APOB *[exon 29], *BRCA1 *[fragment 1], *BRCA1 *[fragment 2], *CHRNA1*, *FES*, *GHR*, *GLB1*, *GNAT1*, *INHBA*, *RHO1*, *TMEM20*, *WT1*); *Mustela nudipes *(*BRCA1 *[fragment 2], *COL10A1*, *FES*, *INHBA*, *TMEM20*); *M. strigidorsa *(*BRCA1 *[fragment 2], *COL10A1*, *TMEM20*); *Procyon lotor *(*TMEM20*); *Vormela peregusna *(*COL10A1*, *FES*, *GLB1*). Question marks were used to represent missing sequences. Despite the amount of missing data for species such as *Melogale personata *and *Mustela altaica*, studies have shown that phylogenetic information content of included data for a taxon is more important in achieving phylogenetic accuracy than the amount of missing data *per se*, especially in the context of a supermatrix analysis [119,120].

### Phylogeny estimation

Gene segments were concatenated into a supermatrix of 11,929 bp, including insertions and deletions (indels). Phylogenetic trees were estimated using MP, ML and BI. Indels were coded as missing for BI and ML analyses (11,929 bp) but were recoded as present or absent (1 or 0), regardless of length, to utilize their potential phylogenetic signal for MP analyses (11,789 bp) [121]. PAUP* 4.0b10 [122] was used to reconstruct MP trees. Characters were equally weighted in heuristic searches using 1,000 random stepwise additions, with one tree held at each step during stepwise addition, tree-bisection-reconnection branch swapping, steepest descent option not in effect, no upper bound for MaxTrees and MulTrees option in effect. Clade support was evaluated by bootstrapping, using 3,000 pseudoreplicates and the same heuristic search conditions as described above except only 100 random stepwise additions were used.

The GTR+I+G model was selected as the best-fitting model of DNA substitution for the 11,929 bp data set, using the Akaike information criterion (AIC) as implemented in Modeltest v3.7 [123]. Under this model, ML heuristic searches were conducted using a hill-climbing algorithm and a genetic algorithm as implemented in the programs TREEFINDER [124] and GARLI [125,126], respectively. For GARLI, we used a random starting tree and default settings for the components of the genetic algorithm. Identical topologies and similar log-likelihood scores were obtained for three separate runs with each program. For both methods of ML analysis, 1,000 bootstrap pseudoreplicates were used to assess the support for the ML topology.

We used MrBayes v3.1.2 [127] for Metropolis-coupled MCMC BI of phylogeny. We performed MCMC runs under the GTR+I+G model of DNA substitution, selected by MrModelTest v2.2 [128] using the AIC. MCMC analyses were performed in which model parameters were linked (uniform model) or unlinked (partitioned model) among the 22 gene segments of the concatenated data set. Two simultaneous independent runs of one cold and three heated MCMC chains and each starting with a different random tree were conducted for 5 × 10^6 ^generations, sampling trees every 500 generations. To ensure consistency of results, we ran analyses for both models a second time (four independent runs for both uniform and partitioned models). The following set of priors were used in all analyses: Dirichlet priors for six substitution rates of the GTR model (1, 1, 1, 1, 1, 1); a Dirichlet prior for base frequencies (1, 1, 1, 1); a uniform prior for the proportion of invariant sites (0, 1); a uniform distribution prior for the shape parameter of the gamma distribution of rate heterogeneity among sites (0, 200); all topologies equally probable; and unconstrained branch lengths with an exponential probability density. Potential scale reduction factors (PSRFs) of 1.00 and an average standard deviation of split frequencies for both simultaneous runs of less than 0.01 indicated that runs had converged on a stationary distribution. In addition, using Tracer 1.3 [129], tracer plots and effective sample size values over 200 for estimates of the posterior distribution of tree likelihood and model parameters also indicated that convergence had been reached and that MCMC chains had mixed well. For each independent run, the first 3,000 trees were discarded as burn-in, leaving 14,002 trees used to construct a 50% majority-rule consensus tree. Internodes with posterior probability values of ≥0.95 were considered well supported.

### Ancestral state reconstruction of biogeography

We reconstructed ancestral areas of mustelids using the likelihood reconstruction method [86,130] implemented in Mesquite v1.12 [131]. Taxa were coded into one of two categorical characters, 0 = Old World (Africa, Eurasia) or 1 = New World (North America, South America) or one of four categorical characters, 0 = Eurasia, 1 = North America, 2 = Africa or 3 = South America. Three taxa have Holarctic distributions, *Gulo gulo*, *Mustela erminea *and *M. nivalis*, while a fourth taxon, *Enhydra lutris*, is distributed along coastal waters of the eastern and northern Pacific Ocean. As these four taxa have distributions that span both Old and New Worlds (two state) or Eurasia and North America (four state), they would be coded as polymorphic (0, 1). However, polymorphic characters cannot be used with the likelihood reconstruction method of Mesquite, so we used fossil evidence to assign the four taxa to one of the categorical characters. *Mustela erminea *and *M. nivalis *were coded as 0 = Old World or Eurasia since the earliest fossil remains of these taxa are found in Eurasia [132,133]. The earliest fossil evidence for the wolverine, *G. gulo*, is found in North America, although Eurasian fossils are almost contemporaneous [134,135]. *Gulo*, however, is either related to or descended from *Plesiogulo*, which originated in Asia in the Late Miocene [136]. Therefore, we coded *Gulo *as 0 = Old World or Eurasia. The earliest fossil remains for *Enhydra *are found in Pleistocene deposits of North America [93,137]. Cladistic analyses suggest that *Enhydra *shares ancestry with the extinct *Enhydritherium*, which immigrated to North America from Eurasia in the Late Miocene [138]. Although there is debate about the exact area of origin of *Enhydra *within North America [93,139], the earliest fossils leading to this lineage are of Old World origin [93] and accordingly, we coded this taxon as 0 = Old World or Eurasia. In addition, *M. frenata *is distributed from North America to South America, with the earliest fossil remains found in the former region [89]. Consequently, we coded this species 1 = North America in the four state analyses. Lastly, fossil evidence suggests that *Mellivora capensis *originated in Africa, although the species also ranges into Eurasia [103]. Therefore, this species was coded 3 = Africa in the four state analysis. Taxa whose exact geographic origins were uncertain (i.e. *E. lutris*, *G. gulo *and *M. capensis*) were coded by the alternative state in separate analyses in order to investigate robustness of the biogeographic reconstructions (e.g. *G. gulo *was coded 1 = New World or North America in two-state and four-state analyses, respectively).

For reconstruction of ancestral areas using likelihood, we used the Markov *k*-state one-parameter model (Mk1) [140], which assumes a single rate of transition between two character states. The rooted topology and branch lengths generated from the ML analyses were used to trace characters. We used the likelihood-ratio test to determine the best estimate of the reconstructed character state at each node, setting the likelihood decision threshold at 2.0. If log-likelihoods of two states differed by 2.0 or more, the state with the lower negative log-likelihood was accepted as the best estimate [130]. Character state reconstructions were considered ambiguous at nodes where log-likelihoods differed by less than 2.0.

### Estimation of divergence times

We estimated divergence times of splits using the Bayesian relaxed phylogenetic approach implemented in BEAST v1.4.2 [52,141]. We assumed a GTR+I+G model of DNA substitution with four rate categories. Uniform priors were employed for GTR substitution parameters (0, 100), gamma shape parameter (0, 100) and proportion of invariant sites parameter (0, 1). The uncorrelated lognormal relaxed molecular clock model was used to estimate substitution rates for all nodes in the tree, with uniform priors on the mean (0, 100) and standard deviation (0, 10) of this clock model. We employed the Yule process of speciation as the tree prior and a UPGMA tree to construct a starting tree, with the ingroup assumed to be monophyletic with respect to the outgroup. Eight fossil calibration points were applied as priors to constrain the age of the following nodes (as numbered in Figures 1 and 2): (i) node 1, 24.0 MYA as the minimum age for crown Mustelidae, based on the earliest known mustelid, *Plesictis*, from the Late Oligocene of Europe [14,18] (but see [25]); (ii) node 2, 10.0 MYA as the minimum age for origin of *Mellivora*, from Late Miocene deposits in South Africa [103]; (iii) node 10, 3.6 MYA as the minimum age for origin of *Lutra*, based on fossils of *Lutra affinis *from the Early Pliocene of Europe [93]; (iv) node 11, 1.0 MYA as the minimum age for the origin of *Aonyx*, based on Pleistocene fossils of this species from Africa [104]; (v) node 16, 5.3 MYA as the minimum age for origin of *Mustela *(including *Neovison*), based on fossils from a number of different species of *Mustela *that appear during the Late Miocene throughout Eurasia [142]; (vi) node 18, 1.8 MYA as the minimum age for the earliest fossil remains of *M. erminea *[132]; (vii) node 28, 1.8 MYA as the minimum age for origin of *Vormela*, based on Pleistocene fossil remains of *V. petenyii *from Europe [143]; and (viii) node 38, 3.3 MYA as the minimum age for origin of the subgenus *Martes*, based on fossils of *Martes wenzensis *from the Late Pliocene of Europe [73] (see also [14]). We conducted additional dating analyses by using minimum fossil constraints (ii)–(viii), but changed the age of crown Mustelidae to a maximum of 28.5 MYA, using the first appearance of the stem taxon *Pseudobassaris *(considered the earliest known taxon of the Procyonidae) as the earliest age of the Mustelidae-Procyonidae split [144] (see also [14]). All fossil constraint priors were set as means of a normal distribution, with a standard deviation of 1.0 MYA. We set the mean of the normal distribution of the root height prior to 24 MYA (assuming *Plesictis *as the earliest fossil mustelid), with a standard deviation of 1.0 MYA. To assess the influence of the root height prior on resulting node ages, we also conducted runs in which age of the root height prior was increased to 28.5 MYA and 33.7 MYA, representing minimum and maximum ages for the Early Oligocene. These ages correspond to the approximate times for the Mustelidae-Procyonidae split [22,144]. Three independent MCMC runs of 10,000,000 steps were performed for each combination of crown group age prior (24 MYA, 28.5 MYA) and root height prior (24 MYA, 28.5 MYA, 33.7 MYA), with parameters logged every 1,000 steps. The Auto Optimize Operators function was enabled to maximize efficiency of MCMC runs. Three independent MCMC runs for each analysis were combined to estimate the posterior distribution of the substitution model and tree model parameters, as well as node ages. Analyses of these parameters in Tracer 1.3 [129] suggested that the number of MCMC steps was more than adequate, with effective sample sizes of all parameters often exceeding 1,000 and Tracer plots showing strong equilibrium after discarding burn-in.

## Note added in proof

After our manuscript had been accepted for publication, multiple bugs were noted with BEAST v1.4.2, which have since been corrected in later versions. One of these bugs did not correctly implement the Yule model of speciation prior, which we used in our divergence time analyses (see methods). This incorrect implementation of the Yule prior could result in biased estimates of divergence times at the root of the phylogenetic tree, particularly when used in conjunction with a relaxed molecular clock model, as we did in our analyses. However, divergence time estimates were probably not significantly biased because we calibrated the height of the root using three different root age priors (see [156]). We nevertheless re-estimated divergence times by conducting three independent MCMC runs using BEAST v1.4.6, the root age and crown age prior combination of 28.5 MYA and 24 MYA, and all other settings identical to those described (see methods). The estimated ages of the root and node 1 (as well the ages of the remaining nodes) from the analyses using BEAST v1.4.2 and BEAST v1.4.6 were highly similar (after discarding burn-in), suggesting that the Yule model bug in BEAST v1.4.2 did not compromise our divergence time estimates. For example, the mean age (and 95% HPD) of the root was estimated as 28.5 MYA (26.6–30.4) and 28.2 MYA (26.3–30.1 MYA) using BEAST v1.4.2 and BEAST v.1.4.6, respectively. The mean age and 95% HPD of node 1 using BEAST v1.4.2 and BEAST v.1.4.6 was 21 MYA (19–23 MYA) and 21.1 MYA (19.1–23.1 MYA), respectively.

## Authors' contributions

KPK conceived of and designed the study, collected the sequence data, performed the phylogenetic analyses, assisted with the dating analyses and drafted the manuscript. KD performed the dating analyses. GJS helped collect the sequence data and performed the historical biogeographic analyses. CB, KB, LG, ML and GV contributed new reagents or analytic tools and also contributed to writing the paper. RKW supervised the study and helped draft the manuscript. All authors read and approved the final manuscript.

## Supplementary Material

Additional file 1 **Leaf stability analysis results**. Three measures of leaf stability (maximum, difference, and entropy) based on MP bootstrap analyses of the 46 taxa data set. Click here for file

Additional file 2 **The prior probability distributions and posterior probability distributions for eight calibration points employed in dating analyses using the program BEAST **[141]. The posterior probability distributions for eight calibration points derived from MCMC analyses without data were run to assess the choice of the joint priors on the posterior estimates when data are included. Click here for file

Additional file 3 **Likelihood values for the reconstruction of ancestral areas**. Proportional likelihood values for the reconstruction of ancestral areas in the two-state and four-state analyses (shown in Figures 3 and 4, respectively). Click here for file

Additional file 4 **Sample origin**. The species, common name and sample origin for taxa sampled. Click here for file

Additional file 5 **Nuclear gene primer information**. Gene symbol and name, primer sequences and description of the 21 nuclear gene segments used in the study. Click here for file

Additional file 6 **Genbank accession numbers**. Genbank accession numbers. Click here for file
