# Supplementary material for: Multigene phylogeny of the Mustelidae: Resolving relationships, tempo and biogeographic history of a mammalian adaptive radiation
Source: BMC Biol. 2008 Feb 14;6:10. doi: 10.1186/1741-7007-6-10 (PMC2276185; doi:10.1186/1741-7007-6-10)
Supplement: Additional file 1 — Leaf stability analysis results. Three measures of leaf stability (maximum, difference, and entropy) based on MP bootstrap analyses of the 46 taxa data set. [file 1741-7007-6-10-S1.doc]

**Additional file 1.** Results of leaf stability analyses. Three measures of leaf stability (maximum, difference, and entropy) based on MP bootstrap analyses of the 46 taxa data set. Average phylogenetic stability shown at bottom of table. Rows shaded gray highlight that *Arctonyx collaris* and *Meles meles* had the lowest leaf stabilities among ingroup taxa.

| Species | MP-Maximum | MP-Difference | MP-Entropy |
| --- | --- | --- | --- |
| *Aonyx capensis* | 0.97 | 0.95 | 0.93 |
| *Aonyx cinerea* | 0.97 | 0.95 | 0.93 |
| *Enhydra lutris* | 0.97 | 0.95 | 0.92 |
| *Lontra canadensis* | 0.97 | 0.95 | 0.93 |
| *Lontra felina* | 0.97 | 0.95 | 0.93 |
| *Lontra longicaudis* | 0.97 | 0.95 | 0.93 |
| *Lutra lutra* | 0.97 | 0.95 | 0.93 |
| *Hydrictis maculicollis* | 0.97 | 0.95 | 0.92 |
| *Pteronura brasiliensis* | 0.97 | 0.95 | 0.92 |
| *Lutra sumatrana* | 0.97 | 0.95 | 0.93 |
| *Lutrogale perspicillata* | 0.97 | 0.95 | 0.93 |
| *Poecilogale albinucha* | 0.96 | 0.93 | 0.89 |
| *Ictonyx libyca* | 0.96 | 0.93 | 0.89 |
| *Vormela peregusna* | 0.96 | 0.93 | 0.89 |
| *Mustela altaica* | 0.97 | 0.95 | 0.93 |
| *Mustela erminea* | 0.97 | 0.95 | 0.93 |
| *Mustela eversmanni* | 0.97 | 0.95 | 0.93 |
| *Mustela frenata* | 0.97 | 0.95 | 0.93 |
| *Mustela lutreola* | 0.97 | 0.95 | 0.93 |
| *Mustela nigripes* | 0.97 | 0.95 | 0.93 |
| *Mustela nivalis* | 0.97 | 0.95 | 0.93 |
| *Mustela nudipes* | 0.97 | 0.95 | 0.93 |
| *Mustela putorius* | 0.97 | 0.95 | 0.93 |
| *Mustela sibirica* | 0.97 | 0.95 | 0.93 |
| *Mustela strigidorsa* | 0.97 | 0.95 | 0.93 |
| *Neovison vison* | 0.97 | 0.95 | 0.93 |
| *Martes americana* | 0.95 | 0.91 | 0.89 |
| *Martes flavigula* | 0.95 | 0.91 | 0.89 |
| *Martes foina* | 0.95 | 0.91 | 0.9 |
| *Martes martes* | 0.95 | 0.91 | 0.9 |
| *Martes melampus* | 0.95 | 0.91 | 0.89 |
| *Martes pennanti* | 0.94 | 0.91 | 0.89 |
| *Martes zibellina* | 0.95 | 0.91 | 0.89 |
| *Gulo gulo* | 0.95 | 0.91 | 0.89 |
| *Eira barbara* | 0.94 | 0.91 | 0.89 |
| *Ictonyx striatus* | 0.96 | 0.93 | 0.89 |
| *Galictis vittata* | 0.96 | 0.93 | 0.89 |
| *Galictis cuja* | 0.96 | 0.93 | 0.89 |
| *Arctonyx collaris* | 0.81 | 0.69 | 0.67 |
| *Meles meles* | 0.81 | 0.69 | 0.67 |
| *Mellivora capensis* | 0.95 | 0.91 | 0.83 |
| *Melogale moschata* | 0.93 | 0.89 | 0.81 |
| *Melogale personata* | 0.93 | 0.89 | 0.81 |
| *Taxidea taxus* | 1 | 1 | 1 |
| *Bassariscus astutus* | 1 | 1 | 1 |
| *Procyon lotor* | 1 | 1 | 1 |
|  |  |  |  |
| **Average** | 0.957 | 0.9287 | 0.902 |
