# Supplementary material for: Multigene phylogeny of the Mustelidae: Resolving relationships, tempo and biogeographic history of a mammalian adaptive radiation
Source: BMC Biol. 2008 Feb 14;6:10. doi: 10.1186/1741-7007-6-10 (PMC2276185; doi:10.1186/1741-7007-6-10)
Supplement: Additional file 2 — The prior probability distributions and posterior probability distributions for eight calibration points employed in dating analyses using the program BEAST [141]. The posterior probability distributions for eight calibration points derived from MCMC analyses without data were run to assess the choice of the joint priors on the posterior estimates when data are included. [file 1741-7007-6-10-S2.doc]

**Additional file 2. Prior probability distributions and posterior probability distributions for eight calibration points employed in dating analyses using the program BEAST [140]. Normal distributions were assumed for prior probability distributions, with a standard deviation of 1.0, with mean and 95% confidence interval values shown as bolded. Posterior probability distributions show mean and 95% highest posterior density values from MCMC runs without data (“No data”) and with data. Posterior distributions from MCMC runs using different combinations of root age and crown age priors are shown.**

|  |  | **24 mya Root Prior** | | **28.5 mya Root Prior** | | | | **33.7 mya Root Prior** | | | |
| --- | --- | --- | --- | --- | --- | --- | --- | --- | --- | --- | --- |
|  |  | **24 mya** | | **24 mya** | | **28.5 mya** | | **24 mya** | | **28.5 mya** | |
| **Calibration node** | **Prior** | **Posterior**  **No data** | **Posterior**  **With data** | **Posterior**  **No data** | **Posterior**  **With data** | **Posterior**  **No data** | **Posterior**  **With data** | **Posterior**  **No data** | **Posterior**  **With data** | **Posterior**  **No data** | **Posterior**  **With data** |
| Root | **-** | 24.5  [22.8-26.1] | 24.2  [22.3-26.0] | 28.5  [26.5-30.4] | 28.5  [26.6-30.4] | 28.9  [27.4-30.7] | 28.6  [26.8-30.5] | 33.7  [31.8-35.6] | 33.6  [31.6-35.5] | 33.7  [31.7-35.7] | 33.7  [31.8-35.7] |
| ***Lutra***  **(node 10)** | **3.6**  **[2.0-5.2]** | 3.2  [1.6-4.9] | 3.7  [2.7-4.6] | 3.2  [1.5-4.9] | 3.7  [2.8-4.7] | 3.2  [1.6-4.9] | 3.6  [2.5-4.7] | 3.2  [1.5-4.9] | 3.7  [2.8-4.7] | 3.2  [1.6-4.9] | 3.6  [2.5-4.8] |
| ***Aonyx***  **(node 11)** | **1.0**  **[-0.6-2.6]** | 1.3  [0.2-2.5] | 2.6  [1.8-3.5] | 1.3  [0.2-2.5] | 2.7  [1.8-3.6] | 1.3  [0.2-2.5] | 2.4  [1.4-3.3] | 1.3  [0.2-2.5] | 2.6  [1.8-3.5] | 1.3  [0.1-2.5] | 2.4  [1.5-3.4] |
| ***Martes***  **(node 38)** | **3.3**  **[1.7-4.9]** | 3.1  [1.5-4.8] | 2.8  [1.9-3.7] | 3.1  [1.5-4.8] | 2.8  [1.96-3.8] | 3.2  [1.5-4.8] | 3.0  [1.8-4.2] | 3.1  [1.4-4.8] | 2.8  [1.9-3.7] | 3.2  [1.5-4.9] | 3.1  [2.1-4.2] |
| ***Mellivora***  **(node 2)** | **10.0**  **[8.4-11.6]** | 10.2  [8.4-12.0] | 12.4  [11.0-13.7] | 10.2  [8.3-12.0] | 12.5  [11.2-13.9] | 10.2  [8.4-12.0] | 12.6  [10.9-14.2] | 10.2  [8.3-12.0] | 12.5  [11.1-14.0] | 10.2  [8.4-12.1] | 12.5  [10.9-14.2] |
| ***Mustela***  **(node 16)** | **5.3**  **[3.7-6.9]** | 4.9  [3.3-6.7] | 6.1  [5.0-7.2] | 4.9  [3.2-6.7] | 6.2  [5.1-7.3] | 5.0  [3.3-6.7] | 6.1  [4.8-7.4] | 4.9  [3.3-6.7] | 6.1  [5.0-7.2] | 5.0  [3.2-6.7] | 6.0  [4.6-7.3] |
| ***Mustela erminea***  **(node 18)** | **1.8**  **[0.2-3.4]** | 2.6  [1.3-4.0] | 3.5  [2.7-4.3] | 2.6  [1.3-4.1] | 3.6  [2.8-4.4] | 2.6  [1.3-4.0] | 3.5  [2.6-4.4] | 2.6  [1.2-3.9] | 3.6  [2.7-4.4] | 2.6  [1.3-4.0] | 3.5  [2.4-4.6] |
| ***Plesictis***  **(node 1)** | **24.0**  **[22.4-25.6]** | 23.2  [21.6-24.9] | 20.9  [18.8-22.9] | 23.7  [21.8-25.7] | 21.0  [19.0-23.1] | - | - | 23.7  [21.8-25.7] | 21.2  [19.3-23.3] | - | - |
| ***Pseudobassaris***  **(node 1)** | **28.5**  **[26.9-30.1]** | - | - | - | - | 27.7  [26.0-29.3] | 26.1  [24.1-28.0] | - | - | 28.2  [26.3-30.3] | 26.2  [24.1-28.2] |
| ***Vormela***  **(node 28)** | **1.8**  **[0.2-3.4]** | 2.0  [0.5-3.4] | 4.5  [3.3-5.8] | 2.0  [0.6-3.5] | 4.6  [3.5-5.85] | 2.0  [0.6-3.5] | 4.0  [2.9-5.3] | 1.9  [0.5-3.4] | 4.6  [3.4-6.0] | 2.0  [0.5-3.4] | 4.0  [2.7-5.4] |
