# Supplementary material for: Multigene phylogeny of the Mustelidae: Resolving relationships, tempo and biogeographic history of a mammalian adaptive radiation
Source: BMC Biol. 2008 Feb 14;6:10. doi: 10.1186/1741-7007-6-10 (PMC2276185; doi:10.1186/1741-7007-6-10)
Supplement: Additional file 3 — Likelihood values for the reconstruction of ancestral areas. Proportional likelihood values for the reconstruction of ancestral areas in the two-state and four-state analyses (shown in Figures 3 and 4, respectively). [file 1741-7007-6-10-S3.doc]

**Additional file 3.** Proportional likelihoods values for reconstruction of ancestral areas in the two state and four state analyses (shown in Figures 3 and 4, respectively). Values in bold indicate best estimates. ? = reconstruction equivocal. ?* = reconstruction equivocal because likelihood ratio test indicated significant likelihood for two or more states. Node numbers correspond to those shown in Figure 1.

|  | **Two State** | | |  | **Four State** | | | | |
| --- | --- | --- | --- | --- | --- | --- | --- | --- | --- |
| **NODE** | Old World | New World | Inferred ancestral area |  | Africa | Eurasia | North America | South America | Inferred ancestral area |
| Root | 81.07 | 18.93 | ? |  | 8.66 | **71.05** | **14.22** | 6.07 | ?* |
| 1 | 84.54 | 15.46 | ? |  | 7.07 | **76.14** | **12.60** | 4.19 | ?* |
| 2 | **93.01** | 6.99 | Old World |  | 5.74 | **86.07** | 5.79 | 2.40 | Eurasia |
| 3 | **96.01** | 3.99 | Old World |  | 1.00 | **97.16** | 1.00 | 0.09 | Eurasia |
| 4 | **96.39** | 3.61 | Old World |  | 0.80 | **97.54** | 0.70 | 0.90 | Eurasia |
| 5 | **94.90** | 5.10 | Old World |  | 1.31 | **95.17** | 0.58 | 2.94 | Eurasia |
| 6 | **96.23** | 5.77 | Old World |  | 1.04 | **95.30** | 0.63 | 3.03 | Eurasia |
| 7 | **89.44** | 10.56 | Old World |  | 1.23 | **91.45** | 0.91 | 6.40 | Eurasia |
| 8 | **89.48** | 10.52 | Old World |  | 1.40 | **91.22** | 1.12 | 6.26 | Eurasia |
| 9 | **99.18** | 0.82 | Old World |  | 1.79 | **96.73** | 0.29 | 1.19 | Eurasia |
| 10 | **99.90** | 0.10 | Old World |  | 0.76 | **99.07** | 0.04 | 0.11 | Eurasia |
| 11 | **99.90** | 0.10 | Old World |  | 2.10 | **97.54** | 0.16 | 0.20 | Eurasia |
| 12 | **99.91** | 0.09 | Old World |  | 0.13 | **99.82** | 0.02 | 0.03 | Eurasia |
| 13 | **99.90** | 0.10 | Old World |  | 0.05 | **99.91** | 0.02 | 0.02 | Eurasia |
| 14 | 0.40 | **96.00** | New World |  | 2.23 | **13.83** | **23.02** | **60.89** | ?* |
| 15 | 0.36 | 99.64 | New World |  | 0.14 | 0.69 | 1.10 | **98.03** | South America |
| 16 | **99.00** | 1.00 | Old World |  | 0.58 | **94.28** | 4.26 | 0.89 | Eurasia |
| 17 | **97.00** | 3.00 | Old World |  | 0.15 | **98.82** | 0.82 | 0.20 | Eurasia |
| 18 | **99.70** | 0.30 | Old World |  | 0.02 | **99.91** | 0.05 | 0.02 | Eurasia |
| 19 | **99.95** | 0.05 | Old World |  | - | **99.99** | - | - | Eurasia |
| 20 | **99.91** | 0.09 | Old World |  | 0.01 | **99.96** | 0.01 | 0.01 | Eurasia |
| 21 | 18.36 | 81.64 | ? |  | 1.20 | **12.24** | **85.32** | 1.20 | ?* |
| 22 | **98.87** | 1.13 | Old World |  | 0.14 | **99.50** | 0.21 | 0.15 | Eurasia |
| 23 | **99.98** | 0.02 | Old World |  | - | **99.99** | - | - | Eurasia |
| 24 | **99.97** | 0.03 | Old World |  | - | **99.99** | - | - | Eurasia |
| 25 | **99.57** | 0.43 | Old World |  | 0.01 | **99.77** | 0.21 | 0.01 | Eurasia |
| 26 | **99.98** | 0.02 | Old World |  | - | **99.99** | - | - | Eurasia |
| 27 | 93.28 | 6.72 | Old World |  | 8.17 | **83.33** | 1.55 | 6.95 | Eurasia |
| 28 | **98.10** | 1.90 | Old World |  | **32.49** | **62.10** | 1.88 | 3.53 | ?* |
| 29 | **99.31** | 0.69 | Old World |  | **89.10** | 9.10 | 0.74 | 0.92 | Africa |
| 30 | **99.56** | 0.44 | Old World |  | **96.22** | 3.03 | 0.34 | 0.41 | Africa |
| 31 | **5.89** | **94.11** | New World |  | 0.96 | 2.77 | 0.80 | **95.46** | South America |
| 32 | **99.48** | 0.52 | Old World |  | 0.10 | **99.69** | 0.10 | 0.11 | Eurasia |
| 33 | **95.88** | 4.12 | Old World |  | 0.75 | **97.55** | 0.86 | 0.84 | Eurasia |
| 34 | **90.32** | 9.68 | Old World |  | 0.49 | **96.07** | 1.47 | 1.97 | Eurasia |
| 35 | **91.25** | 8.75 | Old World |  | 0.35 | **96.82** | 1.50 | 1.32 | Eurasia |
| 36 | **97.05** | 2.95 | Old World |  | 0.09 | **99.75** | 0.32 | 0.28 | Eurasia |
| 37 | **98.72** | 1.28 | Old World |  | 0.04 | **99.75** | 0.10 | 0.09 | Eurasia |
| 38 | **98.80** | 0.20 | Old World |  | 0.02 | **99.94** | 0.02 | 0.02 | Eurasia |
| 39 | **99.92** | 0.08 | Old World |  | - | **99.98** | 0.02 | - | Eurasia |
| 40 | **99.98** | 0.02 | Old World |  | - | **99.99** | 0.01 | - | Eurasia |
| 41 | **98.55** | 1.45 | Old World |  | 0.30 | **99.09** | 0.30 | 0.30 | Eurasia |
