# Supplementary material for: Multigene phylogeny of the Mustelidae: Resolving relationships, tempo and biogeographic history of a mammalian adaptive radiation
Source: BMC Biol. 2008 Feb 14;6:10. doi: 10.1186/1741-7007-6-10 (PMC2276185; doi:10.1186/1741-7007-6-10)
Supplement: Additional file 4 — Sample origin. The species, common name and sample origin for taxa sampled. [file 1741-7007-6-10-S4.doc]

| **Additional file 4.** Species, common name and sample origin for taxa sampled. | | |
| --- | --- | --- |
| **Species** | **Common Name** | **Sample origin^a^** |
| Mustelidae, Lutrinae |  |  |
| *Aonyx capensis* | Cape clawless otter | J. Dallas, UK (no voucher) |
| *Aonyx cinerea* | Asian small-clawed otter | Institute of Zoology, London; #1056 |
| *Enhydra lutris* | Sea otter | J. Bodkin, USA (no voucher) |
| *Hydrictis maculicollis* | Spotted-necked otter | Brookfield Zoo, USA; ISIS 24033 |
| *Lontra canadensis* | North American river otter | Louisiana State University Museum of Natural Science; LSU-MNS 2132 |
| *Lontra felina* | Marine otter | C. Vila, Chile (no voucher) |
| *Lontra longicaudis* | Neotropical otter | University of California, Berkeley, Museum of Vertebrate Zoology; MVZ 4913 |
| *Lutra lutra* | Eurasian otter | J. Ruiz-Olmo, Spain (no voucher) |
| *Lutra sumatrana* | Hairy-nosed otter | H. Sasaki, Vietnam (no voucher) |
| *Lutrogale perspicillata* | Smooth-coated otter | H. Jacques, Tampok Zoo, Cambodia (no voucher) |
| *Pteronura brasiliensis* | Giant otter | Hagenbeck Zoo, Germany; I.D. 1384 |
| Mustelidae, Mustelinae |  |  |
| *Arctonyx collaris* | Hog badger | University of California, Berkeley, Museum of Vertebrate Zoology; MVZ 186562 |
| *Eira barbara* | Tayra | University of New Mexico Museum of Southwestern Biology; MSB 58756 |
| *Galictis cuja* | Lesser grison | Argentina (no voucher) |
| *Galictis vittata* | Greater grison | University of California, Berkeley, Museum of Vertebrate Zoology; MVZ 155226 |
| *Gulo gulo* | Wolverine | University of Alaska Museum; AFTC 4036 |
| *Ictonyx libyca* | Saharan striped polecat | T. Maran, Tallinn Zoo, Estonia (no voucher) |
| *Ictonyx striatus* | Striped polecat | Brookfield Zoo, USA; ISIS 880085 |
| *Martes americana* | American marten | H. Henry, USA (no voucher) |
| *Martes flavigula* | Yellow-throated marten | Zoological Society of San Diego, USA; OR 1560 |
| *Martes foina* | Beech marten | University of Alaska Museum; AFTC 17568 |
| *Martes martes* | Pine marten | University of Alaska Museum; AFTC 17559 |
| *Martes melampus* | Japanese marten | R. Masuda, Japan; MME-G1 |
| *Martes pennanti* | Fisher | E. York, USA (no voucher) |
| *Martes zibellina* | Sable | University of Alaska Museum; AFTC 25274 |
| *Meles meles* | Eurasian badger | E. Geffen, UK (no voucher) |
| *Mellivora capensis* | Honey badger | Republic of South Africa (no voucher) |
| *Melogale moschata* | Chinese ferret-badger | University of California, Berkeley, Museum of Vertebrate Zoology; MVZ 186565 |
| *Melogale personata* | Burmese ferret-badger | Muséum National d’Histoire Naturelle, Paris |
| *Mustela erminea* | Ermine | University of Alaska Museum; AFTC 11756 |
| *Mustela eversmanni* | Steppe polecat | D. Biggins, Mongolia (no voucher) |
| *Mustela frenata* | Long-tailed weasel | Museum of Texas Tech University; TK 23477 |
| *Mustela lutreola* | European mink | V. Sidorovich, Belarus (no voucher) |
| *Mustela nigripes* | Black-footed ferret | S. J. O'Brien, USA (no voucher) |
| *Mustela nivalis* | Least weasel | University of Alaska Museum; AFTC 201 |
| *Mustela nudipes* | Malayan weasel | Muséum National d’Histoire Naturelle, Paris |
| *Mustela putorius* | European polecat | Zoological Society of San Diego, USA; OR 1481 |
| *Mustela sibirica* | Siberian weasel | Zoological Society of San Diego, USA; OR 1584 |
| *Mustela strigidorsa* | Back-striped weasel | Muséum National d’Histoire Naturelle, Paris |
| *Neovison vison* | American mink | Museum of Texas Tech University; TK 29694 |
| *Poecilogale albinucha* | Striped weasel | Durban Natural Science Museum, South Africa; DM 6991 |
| *Taxidea taxus* | American badger | University of New Mexico Museum of Southwestern Biology; MSB 64932 |
| *Vormela peregusna* | Marbled polecat | Muséum National d’Histoire Naturelle, Paris |
| Procyonidae |  |  |
| *Bassariscus astutus* | Ringtail | M. Gompper, USA (no voucher) |
| *Procyon lotor* | Raccoon | University of Alaska Museum; AFTC 10903 |

^a^For samples with no voucher, collector and country of origin of sample are indicated.
