# Supplementary material for: Multigene phylogeny of the Mustelidae: Resolving relationships, tempo and biogeographic history of a mammalian adaptive radiation
Source: BMC Biol. 2008 Feb 14;6:10. doi: 10.1186/1741-7007-6-10 (PMC2276185; doi:10.1186/1741-7007-6-10)
Supplement: Additional file 5 — Nuclear gene primer information. Gene symbol and name, primer sequences and description of the 21 nuclear gene segments used in the study. [file 1741-7007-6-10-S5.doc]

**Additional table 5.** Gene symbol and name, primer sequences, and description of the 21 nuclear gene segments used in the study.

| **Gene symbol** | **Gene name** | **Primers** | **Size (bp)** | **Region** | **Reference** |
| --- | --- | --- | --- | --- | --- |
| *ADORA3* | A3 adenosine receptor | F: ACCCCCATGTTTGGCTGGAA  R: GATAGGGTTCATCATGGAGTT | 340 | exon | Murphy et al. [144] |
| *APOB* | Apolipoprotein B | F: GTGCCAGGTTCAATCAGTATAAGT  R: CCAGCAAAATTTTCTTTTACTTCAA | 933 | exon 26 | Amrine-Madsen et al. [145] |
| *APOB* | Apolipoprotein B | F: GGCTGGACAGTGAAATATTATGAAC  R: AATCAGAGAGTTGGTCTGAAAAA | 301 | exon 29 | Jiang et al. [146] |
| *ATP7A* | ATPase, Cu++ transporting alpha polypeptide | F: TCCCTGGACAATCAAGAAGC  R: AAGGTAGCATCAAATCCCATGT | 638 | exon | Murphy et al. [144] |
| *BDNF* | Brain-derived neurotrophic factor | F: CATCCTTTTCCTTACTATGGTT  R: TTCCAGTGCCTTTTGTCTATG | 548 | exon | Murphy et al. [144] |
| *BRCA1-F1* | Breast and ovarian cancer susceptibility gene  (fragment 1) | F: GCCATGTGGCACARATRCTC  R: CTCTRCTTTCTTGATAAARTCCTCAG | 700 | exon 9 | Lindblad-Toh et al. [147] |
| *BRCA1-F2* | Breast and ovarian cancer susceptibility gene  (fragment 2) | F: TCAAAGCGYCAGTCATTTGC  R: AAWCAGACATGGAGAGAARTC | 746 | exon 9 | Lindblad-Toh et al. [147] |
| *CHRNA1* | Cholinergic receptor, nicotinic, alpha polypeptide 1  precursor | F: GACCATGAAGTCAGACCAGGAG  R: GGAGTATGTGGTCCATCACCAT | 385 | exon/intron | Lyons et al. [148] |
| *COL10A1* | Collagen type X a1 | F: ATTCTCTCCAAAGCTTACCC  R: GCCACTAGGAATCCTGAGAA | 324 | exon | Venta et al. [149] |
| *FES* | Feline sarcoma protooncogene | F: GGGGAACTTTGGCGAAGTGTT  R: TCCATGACGATGTAGATGGG | 463 | exon/intron | Venta et al. [149] |
| *GHR* | Growth hormone receptor | F: CCAGTTCCAGTTCCAAAGAT  R: TGATTCTTCTGGTCAAGGCA | 635 | exon/intron | Venta et al. [149] |
| *GLB1* | B-galactosidase | F: GAATTCTATACTGGCTGGCT  R: CATTCCAATAGGCAAAATTGGT | 209 | exon/intron | Venta et al. [149] |
| *GNAT1* | Rod transducin alpha-subunit | F: AGCACCATCGTCAAGCAGA  R: CTGGATACCCGAGTCCTTC | 445 | exon/intron | Brouillette et al. [150] |
| *INHBA* | Inhibin alpha-subunit | F: CAGTCGCACAGACTTTCCTCAT  R: GCACTCCTCCACGATCATGTTCT | 375 | exon | Jiang et al. [146] |
| *PLCB4* | Phospholipase C, beta 4 | F: GTGAAATTGGAAGCCGAGAT  R: CACCAAGCTCATTTACTTGTGA | 313 | 3'UTR | Murphy et al. [144] |
| *PNOC* | Prepronociceptin | F: GCATCCTTGAGTGTGAAGAGAA  R: TGCCTCATAAACTCACTGAACC | 290 | exon | Murphy et al. [144] |
| *RAG1* | Recombination activating protein 1 | F: GCTTTGATGGACATGGAAGAAGACAT  R: GAGCCATCCCTCTCAATAATTTCAGG | 1079 | exon | Teeling et al. [151] |
| *RAG2* | Recombination activating protein 2 | F: TCATGGAGGGAAAACACCAAA  R: TGCACTGGAGACAGAGATTC | 468 | exon | Murphy et al. [144] |
| *RHO1* | Rhodopsin | F: TACATGTTCGTGGTCCACTT  R: TGGTGGGTGAAGATGTAGAA | 284 | exon/intron | Venta et al. [149] |
| *TMEM20* | Transmembrane 20 | F: TGGGTTTATAGGCCCCAAAG  R: CACGTKGGCACATYRTTA | 596 | exon | Lindblad-Toh et al. [147] |
| *WT1* | Wilms tumor 1 | F: GAGAAACCATACCAGTGTGA  R: GTTTTACCTGTATGAGTCCT | 717 | exon/intron | Venta et al. [149] |
