# Supplementary material for: Multigene phylogeny of the Mustelidae: Resolving relationships, tempo and biogeographic history of a mammalian adaptive radiation
Source: BMC Biol. 2008 Feb 14;6:10. doi: 10.1186/1741-7007-6-10 (PMC2276185; doi:10.1186/1741-7007-6-10)
Supplement: Additional file 6 — Genbank accession numbers. Genbank accession numbers. [file 1741-7007-6-10-S6.doc]

**Additional file 6.** Genbank accession numbers.

| **Species** | ***ADORA3*** | ***APOB* (exon 26)** | ***APOB***  **(exon 29)** | ***ATP7A*** | ***BDNF*** | ***BRCA1* (fragment 1)** |
| --- | --- | --- | --- | --- | --- | --- |
| *Aonyx capensis* | EF987478 | EF472285^a^ | EF486495^a^ | EF987562 | EF987606 | EF472301^a^ |
| *Aonyx cinerea* | EF987479 | EF472286^a^ | AF498107^c^ | EF987563 | EF987607 | EF472302^a^ |
| *Enhydra lutris* | DQ660176^b^ | DQ660190^b^ | AF498108^c^ | EF987564 | DQ660204^b^ | EF472303^a^ |
| *Lontra canadensis* | EF987481 | EF472287^a^ | AF498109^c^ | EF987565 | EF987608 | EF472304^a^ |
| *Lontra felina* | EF987482 | EF472288^a^ | AF498110^c^ | EF987566 | EF987609 | EF472305^a^ |
| *Lontra longicaudis* | EF987483 | EF472289^a^ | AF498111^c^ | EF987567 | EF987610 | EF472306^a^ |
| *Lutra lutra* | EF987484 | EF472290^a^ | AF498112^c^ | EF987568 | EF987611 | EF472307^a^ |
| *Hydrictis maculicollis* | EF987485 | EF472291^a^ | AF498113^c^ | EF987569 | EF987612 | EF472308^a^ |
| *Pteronura brasiliensis* | EF987486 | EF472292^a^ | AF498114^c^ | EF987570 | EF987613 | EF472309^a^ |
| *Lutra sumatrana* | EF987487 | EF472293^a^ | EF472298^a^ | EF987571 | EF987614 | EF472310^a^ |
| *Lutrogale perspicillata* | EF987488 | EF472294^a^ | EF472299^a^ | EF987572 | EF987615 | EF472311^a^ |
| *Mustela altaica* | n.s. | AB193413^e^ | n.s. | n.s. | n.s. | n.s. |
| *Mustela erminea* | EF987492 | EF987522 | AF498115^c^ | EF987575 | EF987619 | EF987646 |
| *Mustela eversmanni* | EF987493 | EF987523 | EF987547 | EF987576 | EF987620 | EF987647 |
| *Mustela frenata* | EF987494 | EF472296^a^ | AF498116^c^ | EF987577 | EF987621 | EF472313^a^ |
| *Mustela lutreola* | EF987495 | EF987524 | EF987548 | EF987578 | EF987622 | EF987648 |
| *Mustela nigripes* | EF987496 | EF987525 | EF987549 | EF987579 | EF987623 | EF987649 |
| *Mustela nivalis* | EF987497 | EF987526 | EF987550 | EF987580 | EF987624 | EF987650 |
| *Mustela nudipes* | EF987498 | EF987527 | EF987551 | EF987581 | EF987625 | EF987651 |
| *Mustela putorius* | EF987499 | EF987528 | EF987552 | EF987582 | EF987626 | EF987652 |
| *Mustela sibirica* | EF987500 | EF987529 | EF987553 | EF987583 | EF987627 | EF987653 |
| *Mustela strigidorsa* | EF987501 | EF987530 | EF987554 | EF987584 | EF987628 | EF987654 |
| *Neovison vison* | DQ660177^b^ | DQ660191^b^ | AF498117^c^ | EF987585 | DQ660205^b^ | EF472314^a^ |
| *Galictis cuja* | EF987514 | EF987539 | EF987560 | EF987597 | EF987638 | EF987665 |
| *Galictis vittata* | EF987513 | EF987538 | EU003532 | EF987596 | EF987637 | EF987664 |
| *Ictonyx libyca* | EF987490 | EF987520 | EF987545 | n.s. | EF987617 | EF987644 |
| *Ictonyx striatus* | EF987512 | EF472297^a^ | AF498122^c^ | EF987595 | EF987636 | EF472315^a^ |
| *Poecilogale albinucha* | EF987489 | EF472295^a^ | EF472300^a^ | EF987573 | EF987616 | EF472312^a^ |
| *Vormela peregusna* | EF987491 | EF987521 | EF987546 | EF987574 | EF987618 | EF987645 |
| *Eira barbara* | DQ660178^b^ | DQ660192^b^ | AF498121^c^ | EF987594 | DQ660206^b^ | EF987663 |
| *Gulo gulo* | EF987510 | EF987537 | AF498120^c^ | EF987593 | EF987635 | EF987662 |
| *Martes americana* | DQ660179^b^ | DQ660193^b^ | AF498118^c^ | EF987586 | DQ660207^b^ | EF987655 |
| *Martes flavigula* | EF987504 | EF987531 | EF987555 | EF987587 | EF987629 | EF987656 |
| *Martes foina* | EF987505 | EF987532 | EF987556 | EF987588 | EF987630 | EF987657 |
| *Martes martes* | EF987506 | EF987533 | EF987557 | EF987589 | EF987631 | EF987658 |
| *Martes melampus* | EF987507 | EF987534 | EF987558 | EF987590 | EF987632 | EF987659 |
| *Martes pennanti* | EF987508 | EF987535 | EF987559 | EF987591 | EF987633 | EF987660 |
| *Martes zibellina* | EF987509 | EF987536 | EF987555 | EF987592 | EF987634 | EF987661 |
| *Arctonyx collaris* | EF987515 | EF987540 | AF498125^c^ | EF987598 | EF987639 | EF987666 |
| *Meles meles* | EF987516 | EF987541 | AF498123^c^ | EF987599 | EF987640 | EF987667 |
| *Mellivora capensis* | n.s. | EF987542 | EF987561 | EF987600 | EF987641 | EF987668 |
| *Melogale moschata* | EF987517 | EF987543 | AF498126^c^ | EF987601 | EF987642 | EF987669 |
| *Melogale personata* | EF987518 | EF987544 | n.s. | EF987602 | EF987643 | EF987670 |
| *Taxidea taxus* | DQ660180 ^b^ | DQ660194^b^ | AF498124^c^ | EF987603 | DQ660208^b^ | EF987671 |
| *Bassariscus astutus* | DQ660169^b^ | DQ660183^b^ | AF498127^c^ | EF987604 | DQ660197^b^ | EF987672 |
| *Procyon lotor* | DQ660175^b^ | DQ660189^b^ | AF498128^c^ | EF987605 | DQ660203^b^ | EF987666 |

| **Species** | ***BRCA1* (fragment 2)** | ***CHRNA1*** | ***COL10A1*** | ***CYTb*** | ***FES*** | ***GHR*** |
| --- | --- | --- | --- | --- | --- | --- |
| *Aonyx capensis* | EF472316^a^ | AF498129^c^ | EF472334^a^ | AF057118^d^ | AF498160^c^ | AF498184^c^ |
| *Aonyx cinerea* | EF472317^a^ | AF498130^c^ | EF472335^a^ | AF057119^d^ | AF498161^c^ | AF498185^c^ |
| *Enhydra lutris* | EF472318^a^ | AF498131^c^ | DQ660225^b^ | AF057120^d^ | AF498162^c^ | AF498186^c^ |
| *Lontra canadensis* | EF472319^a^ | AF498132^c^ | EF472336^a^ | AF057121^d^ | AF498163^c^ | AF498187^c^ |
| *Lontra felina* | EF472320^a^ | AF498133^c^ | EF472337^a^ | AF057122^d^ | AF498164^c^ | AF498188^c^ |
| *Lontra longicaudis* | EF472321^a^ | AF498134^c^ | EF472338^a^ | AF057123^d^ | AF498165^c^ | AF498189^c^ |
| *Lutra lutra* | EF472322^a^ | AF498135^c^ | EF472339^a^ | AF057124^d^ | AF498166^c^ | AF498190^c^ |
| *Hydrictis maculicollis* | EF472323^a^ | AF498136^c^ | EF472340^a^ | AF057125^d^ | AF498167^c^ | AF498191^c^ |
| *Pteronura brasiliensis* | EF472324^a^ | AF498137^c^ | EF472341^a^ | AF057126^d^ | AF498168^c^ | AF498192^c^ |
| *Lutra sumatrana* | EF472325^a^ | EF472331^a^ | EF472342^a^ | EF472347^a^ | EF472350^a^ | EF472353^a^ |
| *Lutrogale perspicillata* | EF472326^a^ | EF472332^a^ | EF472343^a^ | EF472348^a^ | EF472351^a^ | EF472354^a^ |
| *Mustela altaica* | n.s. | n.s. | n.s. | AB026100^f^ | n.s. | n.s. |
| *Mustela erminea* | EF987675 | AB498138^c^ | EF987717 | AF057127^d^ | AF498169^c^ | AF498193^c^ |
| *Mustela eversmanni* | EF987676 | EF987701 | EF987718 | EF987741 | EF987758 | EF987774 |
| *Mustela frenata* | EF472328^a^ | AF498139^c^ | EF472345^a^ | AF498153^c^ | AF498170^c^ | AF498194^c^ |
| *Mustela lutreola* | EF987677 | EF987702 | EF987719 | EF987742 | EF987759 | EF987775 |
| *Mustela nigripes* | EF987678 | EF987703 | EF987720 | EF987743 | EF987760 | EF987776 |
| *Mustela nivalis* | EF987679 | EF987704 | EF987721 | EF987744 | EF987761 | EF987777 |
| *Mustela nudipes* | n.s. | EF987705 | n.s. | EF987745 | n.s. | EF987778 |
| *Mustela putorius* | EF987680 | EF987706 | EF987722 | EF987746 | EF987762 | EF987779 |
| *Mustela sibirica* | EF987681 | EF987707 | EF987723 | EF987747 | EF987763 | EF987780 |
| *Mustela strigidorsa* | n.s | EF987708 | n.s. | EF987748 | EF987764 | EF987781 |
| *Neovison vison* | EF472329^a^ | AF498140^c^ | DQ660226^b^ | AF057129^d^ | AF498171^c^ | AF498195^c^ |
| *Galictis cuja* | n.s. | EF987714 | EF987733 | EF987754 | EF987770 | EF987787 |
| *Galictis vittata* | EF987691 | AB498145^c^ | EF987732 | AF498155^c^ | AF498176^c^ | AF498200^c^ |
| *Ictonyx libyca* | EF987673 | EF987699 | EF987716 | EF987739 | EF987757 | EF987772 |
| *Ictonyx striatus* | EF472330^a^ | AF498146^c^ | EF472346^a^ | AF498156^c^ | AF498177^c^ | AF498201^c^ |
| *Poecilogale albinucha* | EF472327^a^ | EF472333^a^ | EF472344^a^ | EF472349^a^ | EF472352^a^ | EF472355^a^ |
| *Vormela peregusna* | EF987674 | EF987700 | n.s. | EF987740 | n.s. | EF987773 |
| *Eira barbara* | EF987690 | AB498144^c^ | DQ660227^b^ | AF498154^c^ | AF498175^c^ | AF498199^c^ |
| *Gulo gulo* | EF987689 | AB498143^c^ | EF987731 | X94921^g^ | AF498174^c^ | AF498198^c^ |
| *Martes americana* | EF987682 | AB498141^c^ | DQ660228 ^b^ | AF057130^d^ | AF498172^c^ | AF498196^c^ |
| *Martes flavigula* | EF987683 | EF987709 | EF987725 | EF987749 | EF987765 | EF987782 |
| *Martes foina* | EF987684 | EF987710 | EF987726 | EF987750 | EF987766 | EF987783 |
| *Martes martes* | EF987685 | EF987711 | EF987727 | EF987751 | EF987767 | EF987784 |
| *Martes melampus* | EF987686 | EF987712 | EF987728 | EF987752 | EF987768 | EF987785 |
| *Martes pennanti* | EF987687 | AB498142^c^ | EF987729 | AF057131^d^ | AF498173^c^ | AF498197^c^ |
| *Martes zibellina* | EF987688 | EF987713 | EF987730 | EF987753 | EF987769 | EF987786 |
| *Arctonyx collaris* | EF987692 | AB498149^c^ | EF987734 | AF498157^c^ | AF498180^c^ | AF498204^c^ |
| *Meles meles* | EF987693 | AB498147^c^ | EF987735 | X94922^g^ | AF498178^c^ | AF498202^c^ |
| *Mellivora capensis* | EF987694 | EF987715 | EF987736 | EF987755 | EF987771 | EF987788 |
| *Melogale moschata* | EF987695 | AB498150^c^ | EF987737 | AF498158^c^ | AF498181^c^ | AF498205^c^ |
| *Melogale personata* | n.s. | n.s. | EF987738 | EF987756 | n.s. | n.s. |
| *Taxidea taxus* | EF987696 | AB498148^c^ | DQ660229^b^ | AF057132^d^ | AF498179^c^ | AF498202^c^ |
| *Bassariscus astutus* | EF987697 | AB498151^c^ | DQ660218 ^b^ | AF498159^c^ | AF498182^c^ | AF498206^c^ |
| *Procyon lotor* | EF987698 | AB498152^c^ | DQ660224 ^b^ | DQ660306^b^ | AF498183^c^ | AF498207^c^ |

| **Species** | ***GLB1*** | ***GNAT1*** | ***INHBA*** | ***PLCB4*** | ***PNOC*** | ***RAG1*** |
| --- | --- | --- | --- | --- | --- | --- |
| *Aonyx capensis* | EF987789 | EF472356^a^ | EF987861 | EF472386^a^ | EF987933 | EF472401^a^ |
| *Aonyx cinerea* | EF987790 | EF472357^a^ | EF987862 | EF472387^a^ | EF987934 | EF472402^a^ |
| *Enhydra lutris* | EF987791 | EF472358^a^ | EF987863 | EF472388^a^ | DQ660253^b^ | DQ660267^b^ |
| *Lontra canadensis* | EF987792 | EF472359^a^ | EF987864 | EF472389^a^ | EF987935 | EF472403^a^ |
| *Lontra felina* | EF987793 | EF472360^a^ | EF987865 | EF472390^a^ | EF987936 | EF472404^a^ |
| *Lontra longicaudis* | EF987794 | EF472361^a^ | EF987866 | EF472391^a^ | EF987937 | EF472405^a^ |
| *Lutra lutra* | EF987795 | EF472362^a^ | EF987867 | EF472392^a^ | EF987938 | EF472406^a^ |
| *Hydrictis maculicollis* | EF987796 | EF472363^a^ | EF987868 | EF472393^a^ | EF987939 | EF472407^a^ |
| *Pteronura brasiliensis* | EF987797 | EF472364^a^ | EF987869 | EF472394^a^ | EF987940 | EF472408^a^ |
| *Lutra sumatrana* | EF987798 | EF472353^a^ | EF987870 | EF472395^a^ | EF987941 | EF472409^a^ |
| *Lutrogale perspicillata* | EF987799 | EF472354^a^ | EF987871 | EF472396^a^ | EF987942 | EF472410^a^ |
| *Mustela altaica* | n.s. | n.s. | n.s. | n.s. | n.s. | AB109346^h^ |
| *Mustela erminea* | EF987802 | EF987834 | EF987875 | EF987906 | EF987946 | AB109347^h^ |
| *Mustela eversmanni* | EF987803 | EF987835 | EF987876 | EF987907 | EF987947 | EF987973 |
| *Mustela frenata* | EF987804 | EF472368^a^ | EF987877 | EF472398^a^ | EF987948 | EF472412^a^ |
| *Mustela lutreola* | EF987805 | EF987836 | EF987878 | EF987908 | EF987949 | EF987974 |
| *Mustela nigripes* | EF987806 | EF987837 | EF987879 | EF987909 | EF987950 | EF987975 |
| *Mustela nivalis* | EF987807 | EF987838 | EF987880 | EF987910 | EF987951 | EF987976 |
| *Mustela nudipes* | EF987808 | EF987839 | n.s. | EF987911 | EF987952 | EF987977 |
| *Mustela putorius* | EF987809 | EF987840 | EF987881 | EF987912 | EF987953 | EF987978 |
| *Mustela sibirica* | EF987810 | EF987841 | EF987882 | EF987913 | EF987954 | EF987979 |
| *Mustela strigidorsa* | EF987811 | EF987842 | EF987883 | EF987914 | EF987955 | EF987980 |
| *Neovison vison* | EF987812 | EF472369^a^ | EF987884 | EF472399^a^ | DQ660254^b^ | DQ660268^b^ |
| *Galictis cuja* | EF987824 | EF987853 | EF987896 | EF987924 | EF987965 | EF987984 |
| *Galictis vittata* | EF987823 | EF987852 | EF987895 | EF987923 | EF987964 | EF987983 |
| *Ictonyx libyca* | EF987801 | EF987832 | EF987873 | EF987904 | EF987944 | EF987971 |
| *Ictonyx striatus* | EF987822 | EF472370^a^ | EF987894 | EF472400^a^ | EF987963 | EF472413^a^ |
| *Poecilogale albinucha* | EF987800 | EF472355^a^ | EF987872 | EF472397^a^ | EF987943 | EF472411^a^ |
| *Vormela peregusna* | n.s. | EF987833 | EF987874 | EF987905 | EF987945 | EF987972 |
| *Eira barbara* | EF987821 | EF987851 | EF987893 | EF987922 | DQ660255^b^ | DQ660269^b^ |
| *Gulo gulo* | EF987820 | EF987850 | EF987892 | EF987921 | EF987962 | AB109340^h^ |
| *Martes americana* | EF987813 | EF987843 | EF987885 | EF987915 | DQ660256^b^ | DQ660270^b^ |
| *Martes flavigula* | EF987814 | EF987844 | EF987886 | EF987916 | EF987956 | AB109342^h^ |
| *Martes foina* | EF987815 | EF987845 | EF987887 | EF987917 | EF987957 | AB109343^h^ |
| *Martes martes* | EF987816 | EF987846 | EF987888 | EF987918 | EF987958 | AB109344^h^ |
| *Martes melampus* | EF987817 | EF987847 | EF987889 | EF987919 | EF987959 | EF987981 |
| *Martes pennanti* | EF987818 | EF987848 | EF987890 | EF987920 | EF987960 | EF987982 |
| *Martes zibellina* | EF987819 | EF987849 | EF987891 | n.s. | EF987961 | AB109345^h^ |
| *Arctonyx collaris* | EF987825 | EF987854 | EF987897 | EF987925 | EF987966 | EF987985 |
| *Meles meles* | EF987826 | EF987855 | EF987898 | EF987926 | EF987967 | EF987986 |
| *Mellivora capensis* | EF987827 | EF987856 | EF987899 | EF987927 | EF987968 | EF987987 |
| *Melogale moschata* | EF987828 | EF987857 | EF987900 | EF987928 | EF987969 | AB109357^h^ |
| *Melogale personata* | n.s. | n.s. | n.s. | EF987929 | EF987970 | EF987988 |
| *Taxidea taxus* | EF987829 | EF987858 | EF987901 | EF987930 | DQ660257^b^ | DQ660271^b^ |
| *Bassariscus astutus* | EF987830 | EF987859 | EF987902 | EF987931 | DQ660246^b^ | DQ660260^b^ |
| *Procyon lotor* | EF987831 | EF987860 | EF987903 | EF987932 | DQ660252^b^ | DQ660266^b^ |

| **Species** | ***RAG2*** | ***RHO1*** | ***TMEM20*** | ***WT1*** |
| --- | --- | --- | --- | --- |
| *Aonyx capensis* | EF472414^a^ | AF498208^c^ | EF472430^a^ | EF472445^a^ |
| *Aonyx cinerea* | EF472415^a^ | AF498209^c^ | EF472431^a^ | EF472446^a^ |
| *Enhydra lutris* | DQ660280^b^ | AF498210^c^ | EF472432^a^ | DQ660294^b^ |
| *Lontra canadensis* | EF472416^a^ | AF498211^c^ | EF472433^a^ | EF472448^a^ |
| *Lontra felina* | EF472417^a^ | AF498212^c^ | EF472434^a^ | EF472449^a^ |
| *Lontra longicaudis* | EF472418^a^ | AF498213^c^ | EF472435^a^ | EF472450^a^ |
| *Lutra lutra* | EF472419^a^ | AF498214^c^ | EF472436^a^ | EF472451^a^ |
| *Hydrictis maculicollis* | EF472420^a^ | AF498215^c^ | EF472437^a^ | EF472452^a^ |
| *Pteronura brasiliensis* | EF472421^a^ | AF498216^c^ | EF472438^a^ | EF472453^a^ |
| *Lutra sumatrana* | EF472422^a^ | EF472427^a^ | EF472439^a^ | EF472454^a^ |
| *Lutrogale perspicillata* | EF472423^a^ | EF472428^a^ | EF472440^a^ | EF472455^a^ |
| *Mustela altaica* | n.s. | n.s. | n.s. | n.s. |
| *Mustela erminea* | EF987991 | AF498217^c^ | EF988033 | EF988059 |
| *Mustela eversmanni* | EF987992 | EF988016 | EF988034 | EF988060 |
| *Mustela frenata* | EF472425^a^ | AF498218^c^ | EF472442^a^ | EF472457^a^ |
| *Mustela lutreola* | EF987993 | EF988017 | EF988035 | EF988061 |
| *Mustela nigripes* | EF987994 | EF988018 | EF988036 | EF988062 |
| *Mustela nivalis* | EF987995 | EF988019 | EF988037 | EF988063 |
| *Mustela nudipes* | EF987996 | EF988020 | n.s. | EF988064 |
| *Mustela putorius* | EF987997 | EF988021 | EF988038 | EF988065 |
| *Mustela sibirica* | EF987998 | EF988022 | EF988039 | EF988066 |
| *Mustela strigidorsa* | EF987999 | EF988023 | n.s. | EF988067 |
| *Neovison vison* | DQ660281^b^ | AF498219^c^ | EF472443^a^ | DQ660295^b^ |
| *Galictis cuja* | EF988008 | EF988029 | EF988050 | EF988076 |
| *Galictis vittata* | EF988007 | AF498224^c^ | EF988049 | EF988075 |
| *Ictonyx libyca* | EF987989 | EF988014 | EF988031 | EF988057 |
| *Ictonyx striatus* | EF472426^a^ | AF498225^c^ | EF472444^a^ | EF472459^a^ |
| *Poecilogale albinucha* | EF472424^a^ | EF472429^a^ | EF472441^a^ | EF472456^a^ |
| *Vormela peregusna* | EF987990 | EF988015 | EF988032 | EF988058 |
| *Eira barbara* | DQ660282^b^ | AF498223^c^ | EF988048 | DQ660296^b^ |
| *Gulo gulo* | EF988006 | AF498222^c^ | EF988047 | EF988074 |
| *Martes americana* | DQ660283^b^ | AF498220^c^ | EF988040 | DQ660297^b^ |
| *Martes flavigula* | EF988000 | EF988024 | EF988041 | EF988068 |
| *Martes foina* | EF988001 | EF988025 | EF988042 | EF988069 |
| *Martes martes* | EF988002 | EF988026 | EF988043 | EF988070 |
| *Martes melampus* | EF988003 | EF988027 | EF988044 | EF988071 |
| *Martes pennanti* | EF988004 | AF498221^c^ | EF988045 | EF988072 |
| *Martes zibellina* | EF988005 | EF988028 | EF988046 | EF988073 |
| *Arctonyx collaris* | EF988009 | AF498228^c^ | EF988051 | EF988077 |
| *Meles meles* | EF988010 | AF498226^c^ | EF988052 | EF988078 |
| *Mellivora capensis* | EF988011 | EF988030 | EF988053 | EF988079 |
| *Melogale moschata* | EF988012 | AF498229^c^ | EF988054 | EF988080 |
| *Melogale personata* | EF988013 | n.s. | n.s. | n.s. |
| *Taxidea taxus* | DQ660284^b^ | AF498227^c^ | EF988055 | DQ660298^b^ |
| *Bassariscus astutus* | DQ660274^b^ | AF498230^c^ | EF988056 | DQ660287^b^ |
| *Procyon lotor* | DQ660279^b^ | AF498231^c^ | n.s. | DQ660293^b^ |

^a^Sequence from Koepfli et al. [152].

^b^Sequence from Koepfli et al. [153].

^c^Sequence from Koepfli and Wayne [13].

^d^Sequence from Koepfli and Wayne [12].

^e^Sequence from Sato et al. [16].

^f^Sequence from Kurose et al. [33].

^g^Sequence from Ledje and Árnason [154].

^h^Sequence from Sato et al. [15].

n.s. = no sequence
